# Transcript and protein expression decoupling reveals RNA binding proteins and miRNAs as potential modulators of human aging

**DOI:** 10.1186/s13059-015-0608-2

**Published:** 2015-02-22

**Authors:** Yu-Ning Wei, Hai-Yang Hu, Gang-Cai Xie, Ning Fu, Zhi-Bin Ning, Rong Zeng, Philipp Khaitovich

**Affiliations:** CAS Key Laboratory of Computational Biology, CAS-MPG Partner Institute for Computational Biology, 320 Yue Yang Road, Shanghai, 200031 China; University of the Chinese Academy of Sciences, Beijing, 100039 China; Key Laboratory of Systems Biology, Institute of Biochemistry and Cell Biology, Shanghai Institutes for Biological Sciences, Chinese Academy of Sciences, 320 Yue Yang Road, Shanghai, 200031 China; Max Planck Institute for Evolutionary Anthropology, Deutscher Platz 6, Leipzig, 04103 Germany; Skoltech Center for Computational and Systems Biology, Skolkovo Institute for Science and Technology, Skolkovo, 143025 Russia

## Abstract

**Background:**

In studies of development and aging, the expression of many genes has been shown to undergo drastic changes at mRNA and protein levels. The connection between mRNA and protein expression level changes, as well as the role of posttranscriptional regulation in controlling expression level changes in postnatal development and aging, remains largely unexplored.

**Results:**

Here, we survey mRNA and protein expression changes in the prefrontal cortex of humans and rhesus macaques over developmental and aging intervals of both species’ lifespans. We find substantial decoupling of mRNA and protein expression levels in aging, but not in development. Genes showing increased mRNA/protein disparity in primate brain aging form expression patterns conserved between humans and macaques and are enriched in specific functions involving mammalian target of rapamycin (*mTOR*) signaling, mitochondrial function and neurodegeneration. Mechanistically, aging-dependent mRNA/protein expression decoupling could be linked to a specific set of RNA binding proteins and, to a lesser extent, to specific microRNAs.

**Conclusions:**

Increased decoupling of mRNA and protein expression profiles observed in human and macaque brain aging results in specific co-expression profiles composed of genes with shared functions and shared regulatory signals linked to specific posttranscriptional regulators. Genes targeted and predicted to be targeted by the aging-dependent posttranscriptional regulation are associated with biological processes known to play important roles in aging and lifespan extension. These results indicate the potential importance of posttranscriptional regulation in modulating aging-dependent changes in humans and other species.

**Electronic supplementary material:**

The online version of this article (doi:10.1186/s13059-015-0608-2) contains supplementary material, which is available to authorized users.

## Background

Regulation of gene expression is a fundamental process controlling the implementation of genetic information. While a large part of expression regulation takes place in the transcriptional stage, posttranscriptional regulation also plays critical roles in controlling biological processes. Specifically, posttranscriptional regulation has been shown to contribute to the fine-tuning of gene expression in such cellular processes as apoptosis, immune response, inflammation, neuronal differentiation, synaptic plasticity, the cell cycle, and oncogenesis [1-3]. In all these cases, posttranscriptional regulation was exerted by molecular crosstalk between *cis*-acting sequence elements located on the target RNA and *trans*-acting regulatory factors: RNA binding proteins (RBPs) and non-coding RNAs, such as microRNAs (miRNAs) [4,5].

The human genome encodes as many as 500 known and predicted RBPs identified based on the presence of RNA-binding domains [6]. It is known that RBPs exert their function through binding to specific sequence elements located predominantly but not exclusively in the 3′ UTR of the transcript [7], such as AU-rich elements [8]. Exact binding specificity, however, has been studied for fewer than 50 human RBPs to date [6]. At present, most knowledge of RBP binding sites has been obtained through large-scale crosslinking and immunoprecipitation (CLIP) experiments, including high-throughput sequencing of RNA isolated by CLIP (HITS-CLIP) [9], photoactivatable-ribonucleoside-enhanced CLIP (PAR-CLIP) [10], and individual nucleotide resolution CLIP (iCLIP) [11]. Despite the relatively small number of studies focusing on RBPs, these proteins have been shown to play important roles in controlling splicing, polyadenylation, stability, editing, localization, and translational efficiency of RNA transcripts in human tissues and cell lines [5].

Human miRNAs comprise a large family of over 1,800 small noncoding RNAs with a length of 21 to 25 nucleotides [12]. Functionally, miRNAs guide RNA-induced silencing complex (RISC) to target transcripts in conjunction with Argonaut (AGO) RBPs [13]. Target recognition is mediated by partial binding of an miRNA sequence to a complementary region commonly located in the 3′ UTR of a transcript [14]. While in some cases miRNAs have been shown to function as translational activators, in the vast majority of cases they act as posttranscriptional repressors, reducing the stability and/or translational efficiency of the target transcripts [15].

Expression of many genes has been shown to undergo drastic changes during human development and/or aging at both mRNA and protein levels [16]. Posttranscriptional regulation, especially that mediated by miRNAs, has been repeatedly implicated in the control of gene expression changes at specific developmental transitions in a number of species, including humans [17]. Still, the long-term effects of posttranscriptional regulation on organ and tissue development remain largely unexplored. In aging, regulation of mRNA translation has been shown to modulate longevity in a wide range of model organisms, from yeast to mice [18-21]. Despite this, the extent of posttranscriptional regulation in human development and aging and, specifically, its role in uncoupling protein and mRNA expression changes have not yet been investigated.

In this study, we surveyed transcriptome and proteome changes taking place during postnatal development and aging in humans and rhesus macaques, in a specific brain region, the prefrontal cortex (PFC). In both species, we observed substantial decoupling of mRNA and protein levels in aging, but not during the developmental interval. Genes showing increased mRNA/protein abundance decoupling formed expression patterns conserved between humans and macaques and were enriched in functions associated with lifespan regulation and senescence: mammalian target of rapamycin (mTOR) signaling, mitochondrial function and neurodegeneration. All mRNA/protein expression profiles found in human and macaque brain aging could be linked to specific posttranscriptional regulators, RBPs and miRNAs, based on binding site specificity data determined in large-scale CLIP-seq experiments.

## Results

### Age-dependent mRNA and protein expression in human and macaque brains

To determine the role of posttranscriptional regulation in development and aging, we assessed protein expression levels in human and macaque brains using time-series data collected over the species’ lifespans and compared these with mRNA expression levels measured in a largely overlapping set of samples from prior studies (Table S1 in Additional file 1).

**Figure 1:**
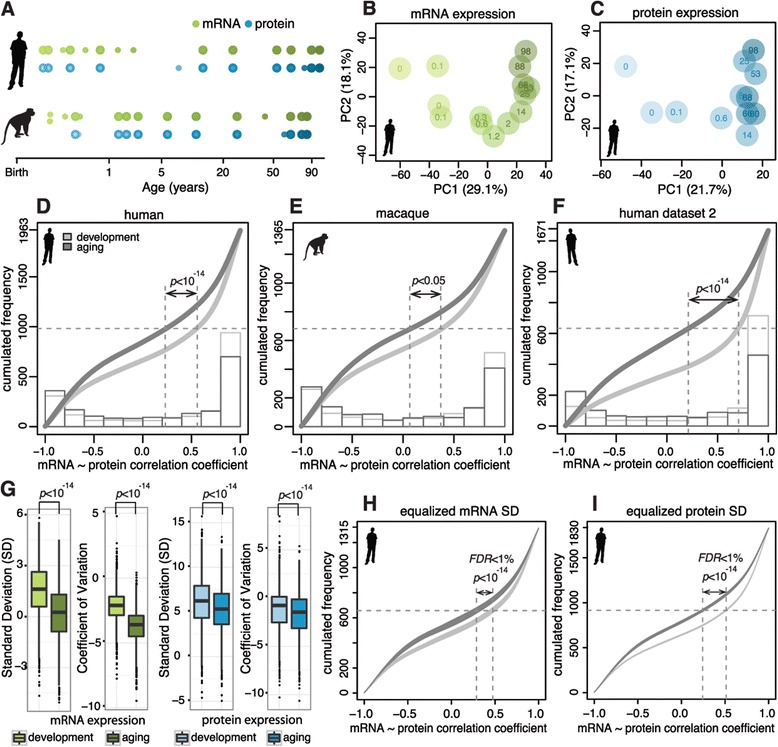
**mRNA/protein decoupling in human and macaque over their lifespans. (A)** Sample age distribution of mRNA (green) and protein (blue) datasets. Each dot represents an individual. Darker shades of color represent older age. Larger dots represent samples used for both mRNA and protein measurements. **(B,C)** The first two principal components of mRNA **(B)** and protein **(C)** expression in human PFC time series. Each circle represents an individual; darker shades of color represent older age; numbers show each individual’s age in years. Proportions of variance explained by each principal component are shown in parentheses. See also Figures S1 and S3 in Additional file 1. **(D-F)** Cumulative frequency of Spearman’s rank correlation coefficients based on mRNA and protein expression changes in developmental (light grey curves) and aging (dark grey curves) intervals. Dashed lines show median values of curves; *P*-values show significance of the difference between medians (Wilcoxon test). Histograms at the bottom of the panels show distributions of Spearman’s rank correlation coefficients in developmental (light grey) and aging (dark grey) intervals composing the curves. Results are shown for human PFC time series **(D)**, macaque PFC time series **(E)** and human PFC time series with another RNA-seq dataset **(F)**. The y-axis shows gene numbers used in each comparison. See also Figure S4 in Additional file 1. **(G)** Box plots show distributions of standard deviation (SD) and coefficient of variation measurements calculated for each gene expressed in human PFC, for developmental (light color) and aging (dark color) intervals, for mRNA (green) and protein (blue) datasets. *P*-values of Wilcoxon tests comparing two distributions are marked above plots. **(H,I)** Distributions of cumulative frequency of Spearman’s rank correlation coefficients based on human mRNA and protein expression changes in developmental (light grey) and aging (dark grey) intervals. Curves are based on 1,000 times subsampling of mRNA and protein expression values. FDR, false discovery rate.

We quantified mRNA expression levels based on high-throughput RNA sequencing (RNA-seq) data for the PFC of 14 humans and 15 rhesus monkeys [22]. These data span most of the species’ lifespans: from 2 days to 98 years in humans and from 1 day to 28 years in macaques (Figure 1A; Tables S2 and S3 in Additional file 1). To quantify and compare gene expression in the two species in an unbiased manner, we mapped human and macaque RNA-seq reads to the consensus genome constructed based on pairwise genome alignment of human and rhesus macaque genomes. This resulted in 213 million (72.4%) human and 323 million (65.7%) macaque uniquely mapped reads. Based on these reads, 11,734 and 12,097 protein-coding genes were classified as expressed in human and macaque time series (Table S4 in Additional file 1).

We measured protein expression levels using label-free quantitative mass spectrometry in PFC samples from 12 humans and 12 rhesus macaques with age distributions mirroring those of the transcriptome measurements: between 1 day and 98 years for humans, and between 16 days and 28 years for macaques (Figure 1A; Tables S5 and S6 in Additional file 1). Of the 12 human samples used for protein measurements, 10 were represented in the mRNA dataset (Tables S2, S3, S4 and S5 in Additional file 1). With the peptide identification false discovery rate (FDR) set to 1%, we identified a total of 754,258 and 689,880 peptides corresponding to 8,011 and 7,708 genes in human and rhesus macaque samples, respectively. Of these genes, 2,278 and 2,351 were reliably detected in at least half of human or macaque samples (Table S7 in Additional file 1).

General analysis of mRNA and protein expression variation among human PFC samples indicated that age explains a substantial proportion of expression differences among samples in both datasets (Figure 1B,C; Figure S1 in Additional file 1). Consistently, of the 11,734 genes expressed in the human PFC, 6,955 showed significant expression changes with age (*P* < 0.05 after Benjamini correction, Fisher’s test). Of these, 1,963 (28.2%) were also detected reliably at the protein level. For these genes, we compared protein and mRNA expression changes with age at 20 time points, interpolated using spline curves fitted to the actual expression data (Figure S2 in Additional file 1). Following other studies, we separated the developmental and aging intervals based on the age of sexual maturity [23,24], with 10 time points interpolated at each of these intervals. Similar procedures were applied to the rhesus macaque age-dependent genes (Figure S3 in Additional file 1) [25,26].

### Increased decoupling of mRNA and protein expression in aging

Substantial influence of posttranscriptional regulation on developmental or aging processes may result in detectable decoupling of mRNA and protein expression changes with age. To measure the concordance of mRNA and protein expression profiles, we used Spearman’s rank correlation coefficients. Results of the correlation analysis differed drastically between the developmental and aging lifespan intervals: the concordance of mRNA and protein expression levels was much higher during development than during aging (*P* < 0.001, Wilcoxon test; Figure 1D-F). The increased decoupling of mRNA and protein expression levels in aging was significant for both the decrease of positive correlations and the increase of negative correlations (*P* < 0.05, FDR <5%, Spearman’s rank correlation) in the aging interval (*P* < 0.05, chi-square test; Figure S4A in Additional file 1).

Increased decoupling of mRNA and protein expression levels in aging was not caused by higher expression variation among human individuals of older age. It was also not caused by a difference in the expression levels or by the amplitude of expression changes between the developmental and aging intervals. Specifically, in our dataset, mRNA and protein expression variation was significantly smaller in the aging than the developmental interval (*P* < 0.001, Wilcoxon test; Figure 1G). Further, subsampling of data using equalized distribution of standard deviation, coefficient of variation, expression levels or expression change amplitudes for developmental and aging intervals did not affect our results (Figure 1H,I; Figure S4B-D in Additional file 1).

**Figure 2:**
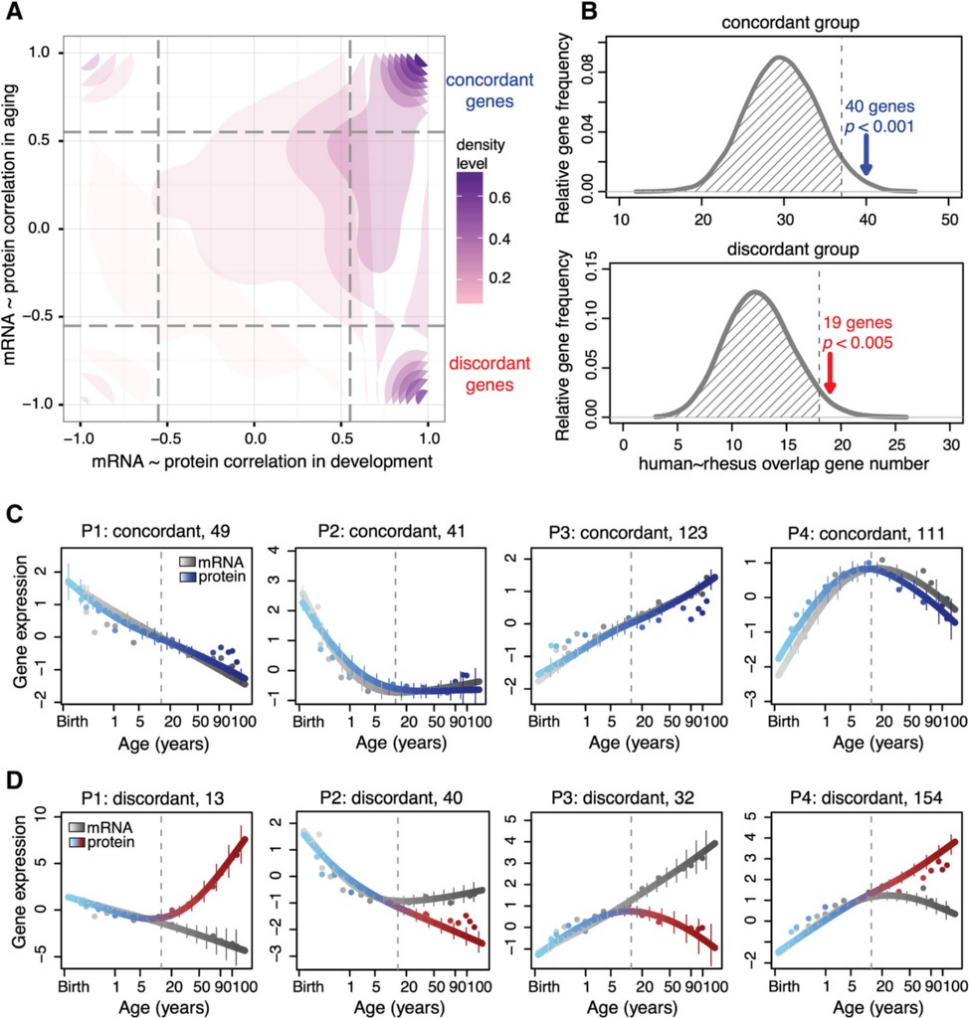
**Concordant and discordant mRNA/protein expression. (A)** Two-dimensional density plot showing distribution of mRNA-protein Spearman’s rank correlation coefficients measured during developmental (x-axis) and aging (y-axis) intervals in human PFC. The grey dashed lines show the correlation coefficient cutoffs used to define concordant and discordant gene groups (*P* < 0.05, Spearman’s rank correlation; FDR <0.05, permutations). See also Figure S5 in Additional file 1. **(B)** The overlap of concordant and discordant gene groups between human and rhesus macaque time series. The arrows show numbers of overlap found in the actual data; the distributions show chance overlap estimated by 1,000 permutations of gene labels. The dashed line indicates the 95% quantile of the distribution. See also Figure S5 in Additional file 1. **(C,D)** Four main patterns of age-dependent mRNA expression separated into concordant **(C)** and discordant **(D)** gene groups. The curves show the average mRNA (gray) and protein (colored) expression calculated using cubic spline regression. The points show the mean expression in each individual. The y-axis shows mRNA and protein expression, normalized to the mean and standard deviation of corresponding expression levels in the developmental interval. The vertical error bars show the standard deviation range of the curves. The pattern number and the number of genes in each group are shown on the top of the panels. The vertical dashed line marks separation of developmental and aging intervals. See also Figure S8 in Additional file 1.

The increased decoupling of mRNA and protein expression levels during aging could be reproduced in the rhesus macaque dataset. Firstly, repeating our analysis based on macaque mRNA and protein time series data, we observed a similar increase in the discordance of mRNA and protein expression changes in the aging interval (Figure 1E; Figure S4E in Additional file 1). Secondly, genes showing concordant and discordant mRNA/protein expression in human aging overlapped significantly with genes showing concordant and discordant expression in macaque aging (*P* < 0.005, permutations) (Figure 2A,B; Figure S5A in Additional file 1). Genes showing concordant and discordant mRNA/protein expression in aging were defined based on a significant positive correlation in the developmental interval and significant positive (concordant genes) or negative (discordant genes) correlations in aging (*P* < 0.05, FDR <5%, Spearman’s rank correlation). In humans, 359 genes were classified as concordant and 260 as discordant (Figure 2A). The fact that increased mRNA-protein disparity could be reproduced in the macaque time series is noteworthy, as the macaque samples were collected from individuals kept in the same standard living conditions and were not subjected to biological and technical artifacts associated with differences in agonal state and post mortem delay.

In addition to the macaque data, increased decoupling of mRNA and protein expression levels during aging could be reproduced using a different published human RNA-seq time series dataset containing data from 38 individuals [27] (Figure 1F; Table S8 in Additional file 1). Comparing human mRNA expression profiles with macaque protein expression profiles and vice versa reproducibly showed greater mRNA/protein expression decoupling in aging (Figure S4F,G in Additional file 1). Furthermore, the concordant and discordant gene groups defined based on any of the above-mentioned comparisons overlapped significantly with corresponding gene groups defined based on the original human dataset (*P* < 0.005, permutations; Figure S5B-G in Additional file 1). Thus, increased decoupling of mRNA and protein expression levels during human and macaque brain aging can be reproducibly observed in multiple datasets and are not likely to be caused by technical artifacts or environmental differences between developmental and aging intervals.

To assess whether increased decoupling of mRNA and protein expression levels in brain aging could be caused by use of different individuals for mRNA and protein measurements or differences in individuals’ ethnicity, we repeated all analyses based on a set of 10 human individuals for which both mRNA and protein data were generated, and based on a subset of 9 of these 10 individuals (all nine being of Caucasian descent). The results of these analyses were in full concordance with those obtained using the full dataset (Figures S6 and S7 in Additional file 1).

### Concordant and discordant genes form conserved co-expressed clusters

To investigate possible functional implications of increased mRNA/protein expression decoupling in human aging, we sorted 359 concordant and 260 discordant genes into co-expressed clusters based on their mRNA expression patterns over the lifespan. A non-supervised hierarchical clustering revealed four main patterns: (P1) mRNA expression continues to decrease across both lifespan intervals; (P2) a decrease in development followed by stabilization or increase in aging; (P3) an increase that continues across the lifespan; and (P4) an increase in development followed by stabilization or a decrease in aging. Within each mRNA co-expression pattern, we then separated the concordant and discordant gene groups (Figure 2C,D). The numbers of genes constituting these gene groups within each pattern varied widely from 11 to 154. Still, for all eight gene groups, the average expression trajectories found in the human data could be reproduced in rhesus macaque time series (Figure S8 in Additional file 1). Thus, the obtained concordant and discordant expression profiles are largely conserved between human and macaque brain development and aging.

Conservation of protein and mRNA co-expression profiles, and their concordant and discordant relationships, suggests this phenomenon is functionally important. Indeed, all eight gene groups show significant enrichment in specific functional terms and pathways specified by Gene Ontology (GO) and Kyoto Encyclopedia of Genes and Genomes (KEGG) annotations [28,29]. While concordant genes are mainly enriched in signal transduction, nucleotide binding and ATP binding, discordant genes show a tendency towards regulatory and signaling functions. Thus, P1 discordant genes are enriched among transcriptional regulators, including zinc finger proteins, and P2 ones in protein binding, kinase activity and translation. Discordant genes in both P2 and P3 are overrepresented in the insulin receptor signaling pathway and mTOR signaling pathway. P4 discordant genes are enriched in mitochondrion, ATP biosynthetic process, calcium ion binding, Huntington’s disease and Parkinson’s disease (Additional file 2).

### Specific RNA binding proteins mediate mRNA and protein expression decoupling in brain aging

Increased decoupling of mRNA and protein expression levels in human brain aging could potentially be caused by actions of common posttranscriptional regulators - RBPs and miRNAs [4,5].

To assess the role of RBPs in increased mRNA/protein disparity, we compiled human transcriptome-wide maps of endogenous binding sites for 17 RBPs from 13 families based on published large-scale CLIP experiments, including HITS-CLIP, PAR-CLIP and iCLIP experimental data [11,30-37] (Table S10 in Additional file 1). To focus on posttranscriptional regulatory effects corresponding to expression level differences between mature mRNAs and proteins, we excluded binding sites located in intronic regions or splice junctions. For each expression pattern, we compared the distributions of RBP binding sites in concordant and discordant gene groups, which differ only by protein expression trajectories in the aging interval. Thus, within the same pattern, differences in RBP binding site distribution should reflect differences in posttranslational regulation between concordant and discordant groups during aging.

**Figure 3:**
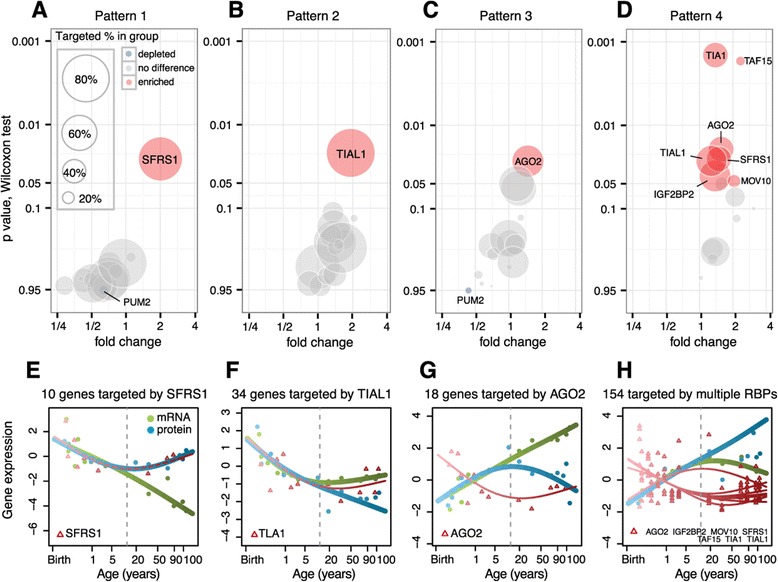
**Regulation of mRNA/protein expression decoupling by RBPs. (A-D)** Enrichment of RBP binding sites within discordant genes in each of the four main expression patterns, compared with concordant genes from the same pattern. The x-axis shows the enrichment fold change based on the binding site number; the y-axis shows the significance of the binding site density difference. Each circle represents one RBP. The circle radius shows the proportion of discordant genes targeted by the RBP within the group. The colors indicate RBPs showing significant enrichment (red), no difference (grey) or significant depletion (blue) of binding sites within discordant genes compared with concordant ones. See also Figure S9 in Additional file 1. **(E-H)** The average mRNA (green) and protein (blue) expression, as well as expression of RBP genes showing significant enrichment of binding sites among discordant genes (red) in each of the four expression patterns. The curves are calculated using cubic spline regression. The symbols show the mean expression in each individual. The y-axis shows mRNA and protein expression, normalized to the mean and standard deviation of corresponding expression levels in the developmental interval. The RBPs and the numbers of target genes in each group are shown on the top of the panels. The vertical dashed line marks separation of the developmental and aging intervals. See also Figure S10 in Additional file 1.

In agreement with our predictions, we found significant enrichment of RBP binding site number and density in discordant gene groups, compared with the concordant groups, for all four expression patterns (Figure 3). Within each discordant group, binding sites corresponding to enriched RBPs covered the majority of genes. Furthermore, the expression patterns of enriched RBPs showed specific correlations with their putative target genes in the corresponding pattern. By contrast, reciprocal analysis of concordant groups yielded no such enrichment of common RBP regulators (Figure S9 in Additional file 1).

Specifically, discordant genes within the P1 pattern show significant enrichment of *SFRS1* binding sites (*P* < 0.05, Wilcoxon test after Bonferroni correction). Notably, among 13 genes in this group, 10 (77%) contained at least one *SFRS1* binding site. Furthermore, *SFRS1* expression significantly correlated positively with protein expression, but not mRNA expression, for 10 putative target genes (*P* < 0.01, Spearman’s rank correlation; Figure 3E; Figure S10 in Additional file 1). This positive relationship is consistent with previous reports demonstrating the role of *SFRS1* as a translational activator in HeLa cells both *in vivo* and *in vitro* [38].

Similarly, discordant genes within the P2 pattern show significant enrichment of *TIAL1* binding sites: as many as 34 (85%) of the 40 genes in this group contain at least one *TIAL1* binding site (*P* < 0.05, Wilcoxon test after Bonferroni correction). In aging, *TIAL1* expression correlated significantly and negatively with protein expression and significantly and positively with mRNA expression for the 34 putative target genes (*P* < 0.01, Spearman’s rank correlation; Figure 3F). Again, this regulatory relationship is consistent with studies reporting that *TIAL1* functions as a translational repressor exhibiting no detectable inhibitory effect at the mRNA level [39].

Genes within the P4 pattern form the largest group, with 154 co-expressed discordant genes. Genes within this group show significant enrichment for seven RBPs: *TAF15*, *MOV10*, *AGO2*, *SFRS1*, *IGF2BP2*, *TIA1* and *TIAL1* (*P* < 0.05, Wilcoxon test after Bonferroni correction). All 154 genes in this group contain at least one binding site for one of the seven enriched RBPs, suggesting that the RBPs examined in our study are, in principle, sufficient to explain the discordant behavior for all genes within this group. With the exception of *TAF15*, expression profiles of the enriched RBPs correlated positively with the protein expression profiles of their putative target genes in the aging interval (Figure 3H).

### Role of miRNAs in mRNA and protein expression decoupling

**Figure 4:**
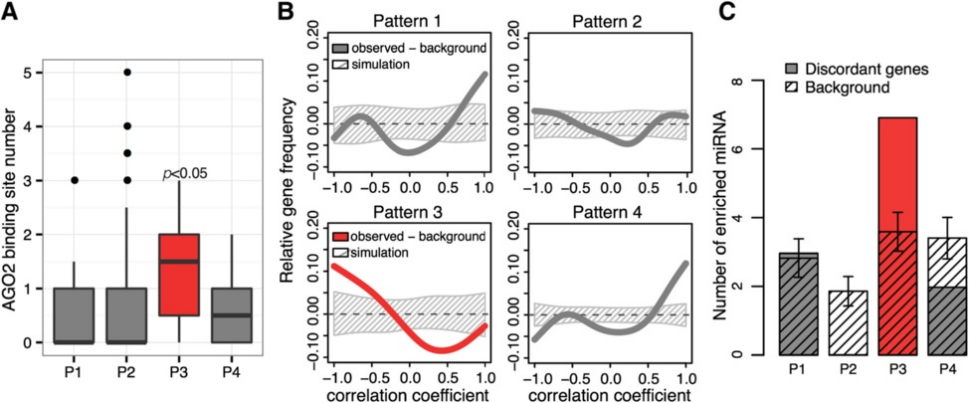
**Regulation of mRNA/protein expression decoupling by miRNAs. (A)** Number of AGO2 binding sites in discordant genes in the four expression patterns. Boxplots show the distribution of AGO2 binding sites in discordant genes and the variation determined by bootstrapping genes within each pattern 1,000 times. The one-sided Wilcoxon test *P*-value shows the significance of the AGO2 binding site excess in P3 compared with the other three patterns. **(B)** Difference between the distribution of predicted miRNA-target correlations and the chance background. The curves show predicted miRNA-target distribution of the Spearman’s rank correlation coefficients measured based on expression profiles of age-dependent miRNA and protein expression of their predicted target transcripts in the aging interval. The background chance distribution was estimated by generating the same number of predicted miRNA-target pairs based on randomly chosen age-dependent miRNA and target genes within the discordant group of each pattern 1,000 times. The shaded areas show the 95% confidence interval of the correlation coefficients’ chance distribution. The median of background chance distribution was subtracted from both the predicted miRNA-target and the chance background distributions. See also Figure S11 in Additional file 1. **(C)** The number of age-dependent miRNAs showing predicted target enrichment among discordant genes of the four patterns. Shaded grey bars show the amount of enriched miRNAs found by generating the same number of predicted miRNA-target pairs based on randomly chosen age-dependent miRNA and target genes within each pattern 1,000 times. See also Figure S12 in Additional file 1.

While decoupling between mRNA and protein expression profiles in human brain aging may, in principle, be driven by RBPs in the P1, P2 and P4 patterns, discordant genes within the P3 pattern show a diverse set of regulatory characteristics. The only RBP showing significant binding site enrichment in the P3 discordant group is AGO2, with 18 (56%) of the 32 genes in this group containing AGO2 binding sites. The number of AGO2 binding sites per gene was nonetheless greater in this group than in the other three discordant groups (Figure 4A). Functionally, AGO2 mediates posttranscriptional regulation as one of the main components of RISC [13]. In agreement with the inhibitory role played by RISC, we see a significant negative correlation between *AGO2* expression and protein expression of its putative target genes in the P3 discordant group (*P* < 0.01, Spearman’s rank correlation; Figure 3G). Specificity of the RISC inhibition, however, is mainly determined by another component of the complex - miRNA [14].

To appraise the possible effects of miRNAs on decoupling mRNA and protein expression profiles in the P3 and other discordant groups, we analyzed published miRNA data collected from the same 12 human individuals used in our proteome analysis [40] (Table S5 in Additional file 1). Of the 373 miRNAs we reliably detected in the dataset, 201 showed significant age-dependent expression level changes (*P* < 0.05, Fisher’s test after Benjamini correction). For these miRNAs, we predicted the locations of conserved target sites, and assessed the correlation between miRNA expression and expression of their predicted target genes in the four discordant groups. Only the P3 discordant group displayed a significant negative correlation between miRNA expression and protein expression of predicted targeted genes in the aging interval (*P* < 0.05 by simulation; Figure 4B). This result was robust to the choice of miRNA target prediction tools using various prediction strategies [41,42] (Figure S11 in Additional file 1). Concordantly, only the P3 discordant group contained a greater than expected number of binding sites for specific miRNAs: out of 12 age-dependent miRNAs showing significant binding site enrichment in any of the four discordant groups, seven were associated with the P3 pattern (*P* < 0.05, hypergeometric test after Bonferroni correction) (Figure 4C; Figure S12 in Additional file 1; Table S11 in Additional file 1). Taken together, these observations suggest that, in contrast to the P1, P2 and P4 expression patterns (where the decoupling of mRNA and protein expression levels in human aging might largely be driven by RPBs alone), mRNA/protein expression decoupling in the P3 pattern may result from cooperative regulation by AGO2 and miRNAs.

## Discussion

In this study, we observed substantial decoupling of mRNA and protein expression profiles during human and rhesus macaque brain aging, but not in the developmental ontogenetic interval. Our results further indicate that all four patterns of aging-dependent mRNA/protein expression decoupling can be associated with a small number of key regulatory RBPs, such as SFRS1, TIAL1 and AGO2. It must be noted that our analyses were limited to experimentally defined RBP targets, mainly identified in cell line experiments. Consequently, many regulatory interactions could have been missed by our analyses. Nonetheless, our results maintain that age-dependent decoupling of mRNA and protein expression patterns could be linked to specific posttranscriptional regulators.

In contrast to the RBP regulatory signal, only one of the four mRNA/protein decoupling patterns characteristic of primate brain aging showed a detectable signature of miRNA-mediated posttranscriptional regulation. This result indicates that the role of RBP-driven regulation of age-dependent changes may be underappreciated at this time.

We observed substantially higher discordance of mRNA and protein expression profiles in aging than in the developmental interval in both humans and macaques. Conservation of age-dependent decoupling profiles between humans and macaques, enrichment of genes following the same decoupling profiles in specific biological processes, as well as association of co-expressed gene groups with specific RBPs and miRNAs, suggest potential functionality of observed posttranscriptional regulation.

One of the strongest signals of RBP-mediated regulation of mRNA/protein discordant profiles that we detected in primate brain aging was TIAL1 association with P2 discordant genes. TIAL1 has been shown to be involved in various forms of translational control [39], including regulation of translation initiation pathways such as the mTOR pathway [43,44]. Notably, P2 discordant genes are significantly enriched in the mTOR pathway (*P* < 0.005, hypergeometric test after Benjamini correction). Conversely, mTOR pathway genes are significantly overrepresented among genes showing discordant expression profiles in primate brain aging (*P* < 0.01, chi-square test). This finding is especially noteworthy, as the mTOR pathway represents one of the few pathways shown to be involved in longevity regulation in a wide range of species [45-48].

**Figure 5:**
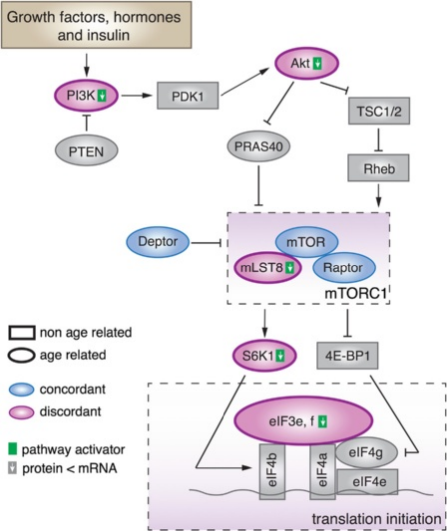
**Discordant and concordant gene expression in the PI3K**-**Akt**-**mTORC1 signaling cascade.** A schematic representation of selected components of the PI3K-Akt-mTORC1 signaling cascade based on [59-62]. The shapes indicate age-dependent (ovals) and non-age-dependent (rectangles) expression of pathway components. The colors indicate concordant (blue) and discordant (rose) expression in the aging interval. For each discordant gene, the green square next to the gene name illustrates its activator function in the pathway, while the white arrow in the square indicates the mRNA-protein expression relationship detected in our data. See also Figure S13 in Additional file 1.

Within the mTOR pathway, all six genes showing discordant expression patterns concentrate within the phosphoinositide 3-kinase (PI3K)-Akt-mTORC1 signaling cascade (Figure 5). This cascade is one of the central, evolutionarily conserved regulators of species’ longevity [49,50]. The best-known function of mTORC1 signaling is the promotion of translation in the presence of extracellular stimuli, such as insulin, hormones and growth factors [51,52]. Extensive studies have shown that reducing activation of the PI3K-Akt signal-inhibiting mTORC1 activity could extend lifespan across multiple species, including mammals [21,53,54]. Moreover, aberrant control of the PI3K-Akt regulatory axis or overactivation of mTOR has been suggested to play a causal role in many aging-related disorders, including cancer, type 2 diabetes mellitus, heart disease and neurodegeneration [50,55,56]. Our study, as well as studies conducted in rats and mice, shows that *mTOR* expression tends to increase with age [57,58]. On the other hand, mTOR activity was shown to decrease with age in the mouse hippocampus due to decreased activity of upstream signaling by PI3K-Akt [58].

Notably, all six genes showing discordant expression patterns within the PI3K-Akt-mTORC1 signaling cascade - *PI3K*, *Akt*, *mLST8*, *S6K1*, *eIF3e* and *eIF3f* - were shown to function as mTOR pathway activators [59-62]. Furthermore, in our data, all of these genes showed the same discordant pattern - translational inhibition in primate brain aging (Figure S13 in Additional file 1).

Of genes showing discordant expression patterns within the PI3K-Akt-mTORC1 signaling cascade, several were directly implicated in lifespan regulation. For example, inhibition of *PI3K* and *Akt* or mutations in *S6K1* have been shown to extend lifespan in yeast, nematodes, fruit flies and mice [21,53,54,63-67]. Similarly, mutation or knockdown of eIFs (translation initiation factors) have been shown to extend lifespan in yeast and nematodes [19,68].

It has been proposed that a global reduction of mRNA translation could promote healthy aging, potentially by allowing endogenous protein repair and degradation machinery to maintain protein homeostasis in the face of protein damage and aggregation [69]. Furthermore, a general reduction in mRNA translation has been suggested to attenuate aging-related pathologies resulting from misplaced activity of biosynthetic and proliferative processes that are important in development but detrimental in aging [70]. Supporting this notion, the PI3K-Akt-mTORC1 pathway has been validated as a potential target of cancer treatment [71]. Notably, the cancer-prevention effect was achieved by repression of the PI3K-Akt-mTORC1 activity through *TIAL1*-mediated translational suppression [72]. Thus, the posttranscriptional regulation signals detected within the PI3K-Akt-mTORC1 pathway in our study may reflect an adaptive organismal response to increased accumulation of molecular damage in advanced age.

Another effect of mTORC1 dysfunction shown in a variety of species, from yeast to mice, is translational activation of mitochondrial genes and enhancement of mitochondrial respiration [46,73,74]. P4 discordant pattern-containing genes showing translational activation in primate brain aging are significantly enriched in mitochondria: as many as 37 of the 154 P4 discordant genes are nuclear-encoded mitochondrial proteins (*P* < 0.05, hypergeometric test after Benjamini correction; Additional file 2). Among others, these genes include the mitochondrial translation initiator *mtIF2*, the mitochondrial translation elongation factor *mtEF-Tu* and six components of the mitochondrial respiration chain. Reduction of bioenergetic efficiency and respiration efficacy mediated by mitochondria was shown to be one of the hallmarks of aging conserved across species [75,76]. Our data, showing translational activation of mitochondrial genes in primate brain aging, suggest that posttranscriptional regulation may play a role in augmenting aging-related decline of mitochondrial functionality. Supporting this notion, translational activation of mitochondrial genes mediated by mTORC1 inhibition has been shown to extend lifespan in yeast and fruit flies [77-79].

The P3 discordant group contains another example of posttranscriptional regulatory signaling, potentially linked to aging-related physiological changes taking place in the primate brain. Enrichment of AGO2 binding sites, combined with a significant negative relationship between miRNA expression and expression of their predicted target proteins, indicates that P3 discordant genes might be subject to miRNA-mediated translational repression. Notably, miRNAs linked with P3 discordant genes overlapped significantly with miRNAs showing aberrant expression in the prefrontal cortex of late-onset Alzheimer’s disease patients (*P* < 0.001, chi-square test) [80]. Furthermore, one of the overlapping miRNAs, miR-132-3p, has been suggested to contribute to Alzheimer’s disease progression through aberrant regulation [81,82]. Interestingly, mir-132-3p has been shown to be regulated by insulin signaling through the PI3K-Akt-mTORC1 cascade [83]. Expression of some miRNAs linked with P3 discordant genes is increased in healthy aging, resulting in posttranscriptional inhibition of predicted P3 target genes (Figure S12 in Additional file 1). Interestingly, in Alzheimer’s disease patients, expression of these miRNAs is decreased compared with healthy aging [80]. This suggests that impaired miRNA-mediated posttranscriptional inhibition of P3 genes might be one of the important features of Alzheimer’s disease.

## Conclusions

Our results indicate that increased decoupling of mRNA and protein expression profiles reproducibly detected in human and macaque brain aging can be linked to specific posttranscriptional regulators - RBPs and miRNAs. Genes targeted and predicted to be targeted by the aging-dependent posttranscriptional regulation can be associated with biological processes known to play important roles in aging and lifespan extension, such as mTOR pathway, mitochondrial function and Alzheimer’s disease. The directions of aging-dependent expression changes observed at the protein level further suggest the potential role of posttranscriptional regulation in counteracting the effects of aging decline. Taken together, these results indicate the potential importance of RBP-mediated posttranscriptional regulation in controlling progression of the human aging phenotype.

## Materials and methods

### Ethics statement

Informed consent for the use of human tissues for research was obtained in writing from all donors or their next of kin. All macaques used in this study suffered sudden deaths for reasons other than their participation in this study and without any relation to the tissue used. The Biomedical Research Ethics Committee of Shanghai Institutes for Biological Sciences reviewed and approved the use and care of the animals in this research (approval ID: ER-SIBS-260802P).

### Sample collection

We collected superior frontal gyrus samples from post-mortem brains of healthy humans and macaques (Tables S5 and S6 in Additional file 1; for details, see supplemental experimental procedures in Additional file 1).

### Protein sample preparation and label-free two-dimensional tandem mass spectrometry

We followed the procedure of protein sample preparation and two-dimensional liquid chromatography coupled with tandem mass spectrometry (LC-MS/MS) analysis described in [84] in a pH continuous online gradient (pCOG) system. Briefly, proteins were extracted from 100 mg of frozen prefrontal cortex tissue from 12 humans and 12 macaques (Tables S5 and S6 in Additional file 1). We extracted the protein samples and incubated them overnight with trypsin, followed by ultrafiltration and lyophilization. Lyophilized protein samples were then loaded on ion exchange columns and eluted using a pH continuous gradient buffer for the LC-MS/MS analysis (for details, see supplemental experimental procedures in Additional file 1).

### Pre-processing of RNA deep sequencing, miRNA deep sequencing and quantitative proteomics data

#### Human-macaque consensus reference genome construction

Chained and netted alignment files for human (hg19) and macaque (rheMac3), aligned using BLASTZ [85], were downloaded from the UCSC Genome Browser. We used the human genome as a reference for all discordant genomic sites, and replaced insertions and deletions with 'N's in the consensus genome. Furthermore, 6 bp regions flanking each insertion or deletion site were masked by 'N's in the resulting human-macaque consensus reference genome (for details, see supplemental experimental procedures in Additional file 1).

#### Computational pre-processing of mRNA deep sequencing

The deep sequencing data of human and rhesus superior frontal gyrus of the PFC were obtained from the Sequence Reads Archive [86] under the accessions SRP005169 for [22] (Tables S1, S2 and S3 in Additional file 1).

To quantify and compare gene expression in the two species in an unbiased manner, we mapped human and rhesus RNA-seq reads to the human-macaque consensus reference genome, using STAR (v.2.3.0e) [87]. Potential PCR duplicates were removed, and only uniquely mapped reads were used in further analyses [88].

Gene expression was quantified as the number of reads per kilobase per million of total mapped reads (RPKM) by GENCODE annotation (v.17) [89,90].

Only genes with RPKM ≥1 in more than two-thirds of the samples in one species (human or macaque) were classified as reliably expressed in that species and were used in the following analysis (for details, see supplemental experimental procedures in Additional file 1).

#### Computational pre-processing of quantitative proteomics

Peptides were identified by searching spectrums against the UniProt Knowledgebase (UniProKB) complete proteome human set [91], using the database search engine MS-GF+ [92]. We estimated the peptide-spectrum matches (PSM) level FDR of peptide identification by searching the combined database of the target dataset and the reversed decoy database. Only peptides with FDR <1% were considered. Ten MS/MS scans of each sample were combined together.

Peptide data were mapped per gene to Ensembl genes by UniProtKB/Swiss-Prot and UniProtKB/TrEMBL annotations. Criteria for protein identification included detection of at least two unique peptides. Ambiguous peptides and redundant proteins were removed.

Quantification of protein expression of each gene was achieved by the normalized spectral abundance factor (NSAF) [93]. The counts of MS/MS spectra assigned to a protein were normalized to the length of the protein, resulting in a spectral abundance factor (SAF). Each SAF was further normalized against the sum of all SAFs in one sample, resulting in the NSAF value.

Only genes with a mean NSAF ≥1 and a positive NSAF value in at least 6 of the 12 individuals were classified as reliably detected in a species (human or macaque), and were included in the downstream analysis (for details, see supplemental experimental procedures in Additional file 1).

#### Computational pre-processing of miRNA deep sequencing

The miRNA deep sequencing data for human PFC were obtained from the NCBI Gene Expression Omnibus [94] under series accession number GSE18069 for [40] (Tables S1, S2, S3, S4 and S5 in Additional file 1).

We followed the procedures of small RNA sequencing data pre-processing and miRNA expression quantification from [95]. All unique sequences were trimmed to remove the adapter sequence at the 3′ end. Trimmed sequences were mapped to the human genome (hg19) by the Bowtie algorithm [96], requiring a perfect match. We quantified miRNA expression by miRBase version 17 [97] with all perfect mapped sequences. The expression level of each miRNA was calculated as transcripts per million reads (TPM; the number of reads mapped to the transcript normalized by the number of total mapped reads; for details, see supplemental experimental procedures in Additional file 1).

### Computational pre-processing of age-dependent genes

We followed the steps of [98] to test the effect of age on mRNA expression level, using polynomial regression models. We used the power of 0.25 of donor age (for details, see supplemental experimental procedures in Additional file 1) to simultaneously capture developmental- and aging-dependent changes. For each detected gene, we chose the best regression model with scaled age as predictor and mRNA expression level as response, using families of polynomial regression models and the 'adjusted r^2^' criterion. The significance of the chosen regression model was estimated using the F-test, and Benjamini-Hochberg correction was carried out for all tested genes as multiple test correction (for details, see supplemental experimental procedures in Additional file 1).

### Analysis of mRNA-protein disparity

To analyze the correlation between transcriptome and proteomics in the classical framework of human lifespan, considering that our samples’ ages were not uniform along the age range and that the mRNA and protein datasets had different age windows, we interpolated 10 uniform points along the age range of each period, at the 0.25-powered age scale [24,99,100].

Spearman’s rank correlation coefficient rho was calculated for each gene for development and aging periods, respectively, between mRNA and protein expression levels, based on interpolated points of spline curves (for details, see supplemental experimental procedures in Additional file 1).

### Equalization of expression and amplitude

We simulated the distribution of the common part of the distributions of the developmental and aging intervals by average expression levels as well as by gene expression change amplitudes. Then, according to this distribution, we subsampled 1,000 times from age-dependent genes, for each species. In each subsampling, we compared the median of correlation distributions between developmental and aging intervals by Wilcoxon test (for details, see supplemental experimental procedures in Additional file 1).

### Overlap of concordant/discordant gene groups between databases

We calculated the overlap of concordant and discordant gene groups resulting from the original human time series dataset, with gene groups resulting independently from (1) a macaque time series dataset, (2) a different human RNA-seq time series dataset, (3) human mRNA and macaque protein expression, and (4) vice versa. We subsampled 1,000 times from all the age-dependent genes of two datasets. In each subsampling, we selected genes from each dataset of the same number as concordant/discordant gene groups and checked the number of overlapped genes, and compared real overlap amount with simulated overlap distribution.

### Clustering genes into groups

We grouped concordant and discordant genes into four patterns of mRNA expression using k-means clustering. Before clustering, each gene was standardized to mean = 0 and standard deviation = 1. Because k-means clustering is a heuristic algorithm, we repeated the procedure 1,000 times to determine the most frequent constellation and used this clustering in downstream analysis.

### Functional analysis

We used the GO categories of biological process (BP), molecular function (MF) and cellular component (CC), together with KEGG pathway databases [28,29], for testing the functional enrichment of gene groups. To identify over-representation, we used GeneCoDis3, a non-redundant and modular enrichment analysis tool for functional annotation of gene sets [101] to investigate the putative functions of concordant and discordant gene groups in each of four patterns. Validated detected age-dependent genes were taken as background, and hypergeometric test *P*-values were adjusted for multiple testing by the Benjamini-Hochberg correction.

### Posttranscriptional regulator RNA binding protein identification

#### Extraction of exonic RNA binding protein binding sites

All data sets from CLIP experiments (including HITS-CLIP, PAR-CLIP and iCLIP) used in the analysis were collected from the published literature (Table S10 in Additional file 1).

The precise positional RBP target sites extracted from the CLIP data sets were mapped to gene locations by Ensembl gene annotation. We followed the cutoff used in the original literature of each CLIP experiment. Only binding sites passing the cutoff were used in the following analysis. To focus on post-transcriptional regulation corresponding to the mature mRNA, we excluded the binding sites located in intronic regions or in intron/exon splicing junctions.

#### Key regulator RNA binding protein identification

To define a RBP as a key regulator of the disparity gene group of a pattern, we checked three conditions: (1) the regulator should target (has at least one validated binding site on the gene) a significantly higher percentage of discordant genes compared with concordant genes in the same pattern. The significance is indicated by *P* < 0.05 in a binomial test after Bonferroni correction between percentages of targeted genes in concordant and discordant gene groups. (2) The regulator should have significantly more binding sites on the exon union of discordant genes compared with concordant genes in the same pattern. The binding site number enrichment is classified with *P* < 0.05 of one-sided Wilcoxon test after Bonferroni correction between distributions of binding site number on genes in concordant and discordant gene groups. (3) After correction by the length of exon union for each gene, the regulator should have significantly higher density of binding sites in discordant genes compared with concordant genes in the same pattern. The significance is indicated by *P* < 0.05 of one-sided Wilcoxon test after Bonferroni correction between distributions of binding site density of genes in concordant and discordant gene groups. One regulator is classified as a key regulator if it fulfils condition 1 and at least one of conditions 2 and 3.

### Posttranscriptional miRNA regulation estimation

#### miRNA binding site extraction from in silico prediction

Human miRNA target prediction was based on miRNA target sites identified using Targetscan [102]. Furthermore, only target sites that were conserved in at least three out of four species (mouse, rat, dog and chicken) were classified as reliable conserved targets [103] (for details, see supplemental experimental procedures in Additional file 1).

#### Enriched targeting miRNA identification

To identify miRNA with enrichment of predicted targets in a discordant gene group in each of the four patterns, we compared miRNA binding site number with concordant genes in the same pattern. To avoid the influence of miRNA gene families, we restricted this comparison to a single test per unique miRNA seed. If a miRNA’s predicted targets were enriched in a pattern after hypergeometric test (*P* < 0.05 after Bonferroni correction), we considered that miRNA 'specific' to that discordant gene group. All the other miRNAs were considered 'non-specific' between the concordant and discordant gene groups in this pattern. These results were compared to chance distributions obtained using 1,000 permutations by randomized labeling of concordant/discordant genes within each pattern.

#### Functional miRNA regulation estimation

To estimate the negative effect of miRNAs on the translation of discordant genes in each pattern, we calculated the predicted miRNA-target distribution of the Spearman’s rank correlation coefficients measured based on expression profiles of age-dependent miRNA and protein expression of their predicted target transcripts in aging interval.

The background chance distribution was generated by the same number of predicted miRNA-target pairs based on randomly chosen age-dependent miRNA and target genes within the discordant group of each pattern for 1,000 times.

We estimated the difference between predicted miRNA-target distribution and background chance distribution by subtraction of the median of background chance distribution. A discordant group of one pattern was considered negatively regulated by miRNAs on the translation level if, and only if, significant excess of negative correlation (*P* < 0.05, FDR <5%, Spearman’s rank correlation) was observed. The significance is indicated by 95% confidence intervals of chance difference obtained in background chance distribution.

### Data availability

All proteomics data have been deposited in the public PeptideAtlas database under accession number PASS00505.

## Additional files

Additional file 1:
**Figures S1 to S13 and legends, supplemental Tables S1 to S8 and S10 and S11, and a complete and comprehensive list of experimental procedures and supplemental references.**
Additional file 2:
**Table S9, which lists GO functional terms and KEGG pathways enriched in concordant and discordant genes.**

## Abbreviations

AGO: Argonaut
CLIP: crosslinking and immunoprecipitation
FDR: false discovery rate
GO: Gene Ontology
HITS-CLIP: high-throughput sequencing of RNA isolated by CLIP
iCLIP: individual-nucleotide resolution CLIP
KEGG: Kyoto Encyclopedia of Genes and Genomes
LC: liquid chromatography
miRNA: microRNA
MS/MS: tandem mass spectrometry
mTOR: mammalian target of rapamycin
NSAF: normalized spectral abundance factor
PAR-CLIP: photoactivatable-ribonucleoside-enhanced CLIP
PFC: prefrontal cortex
PI3K: phosphoinositide 3-kinase
RBP: RNA binding protein
RISC: RNA-induced silencing complex
RPKM: reads per kilobase per million of total mapped reads
SAF: spectral abundance factor
UTR: untranslated region
