# Supplementary material for: Transcript and protein expression decoupling reveals RNA binding proteins and miRNAs as potential modulators of human aging
Source: Genome Biol. 2015 Feb 22;16(1):41. doi: 10.1186/s13059-015-0608-2 (PMC4375924; doi:10.1186/s13059-015-0608-2)
Supplement: Additional file 1: — Figures S1 to S13 and legends, supplemental Tables S1 to S8 and S10 and S11, and a complete and comprehensive list of experimental procedures and supplemental references. [file 13059_2015_608_MOESM1_ESM.pdf]

# Supplemental Figures

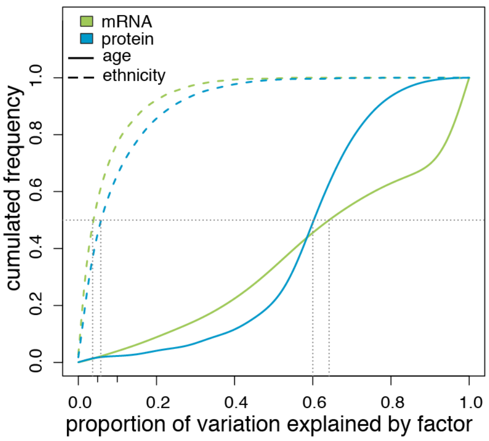


**Figure S1. Effect of age and ethnicity on expression of human datasets.**

Cumulative frequency curves showing the proportions of mRNA and protein expression variance explained by age (solid lines) and ethnicity (dashed lines) across genes expressed in the human mRNA (green) and protein (blue) datasets. The proportions were calculated using polynomial regression models. The grey dotted lines show median proportions of the variance explained by the factors: 3% of mRNA and 6% of protein expression variation – by ethnicity; and 64% of mRNA and 61% of protein expression variation – by age.

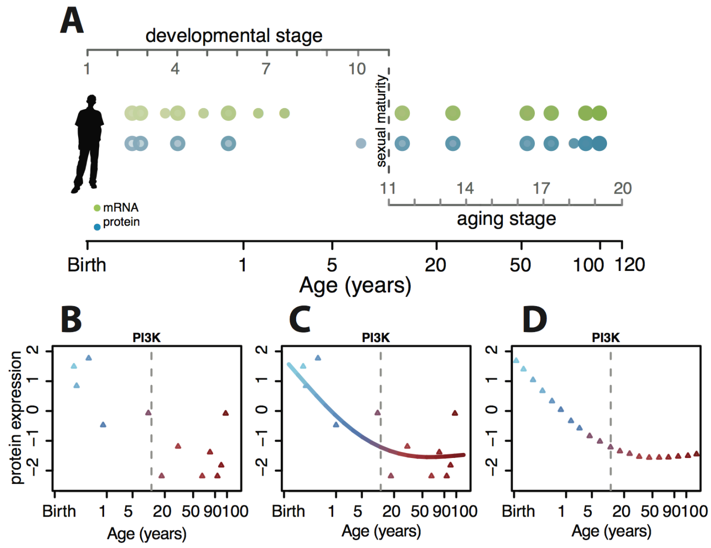


**Figure S2. Illustration of interpolated points of expression levels in human.**

(A) Distributions of actual and interpolated data points along human lifespan. The dashed line shows the age of sexual maturity (here – 14 years for humans, 4 years was used for macaques). Grey horizontal bars show the distribution of 20 time points uniformly spread respectively over developmental (points 1-10) and aging (points 11-20) stages of ontogenesis. The colored dots represent individuals included in mRNA (green) and protein (blue) datasets. The darker shades of

color represent older age. The larger dots represent samples used in both mRNA and protein expression measurements. (B-D) An example of protein expression values interpolation for *PI3K* gene. (B) The symbols show actual protein expression levels in each individual. (C) The curve was calculated using cubic spline regression to the individual protein expression values. (D) Expression levels were interpolated using a spline curve at time points distributed as shown in panel A.

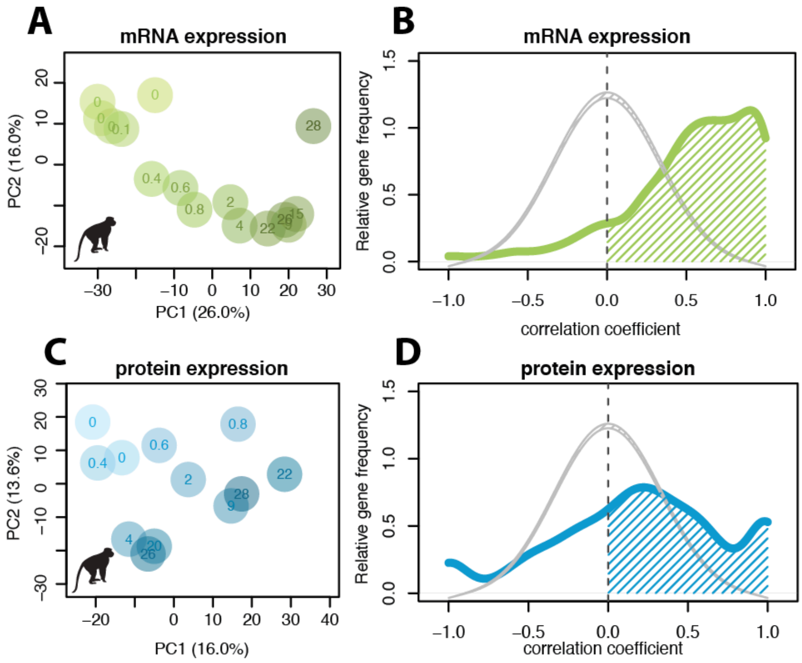


**Figure S3. Age explains a substantial proportion of expression differences among samples in both datasets of rhesus macaque.**

The first two principal components of mRNA (A) and protein (C) expression in macaque PFC samples of different ages. Each circle represents an individual; green colors show mRNA, blue show protein expression. Darker shades of color represent older age; the numbers show each individual's age in years. The proportion of variance explained by each principal component is shown in parentheses.

Distribution of Spearman's rank correlation coefficients between human and macaque time-series data, calculated for 1,609 orthologous genes of both mRNAs (B) and proteins (D), out of 1,963 genes showing significant expression changes with age in humans and reliably detected in macaques. Shaded grey curves show the chance distribution generated by 1,000 permutations of ages. The median values of both simulations of mRNA and protein are 0.0 (shown by a grey dashed line), and are significantly less positively correlated compared to real data (shaded colored part; $p < 0.001$, Wilcoxon test, FDR < 0.001, permutation).

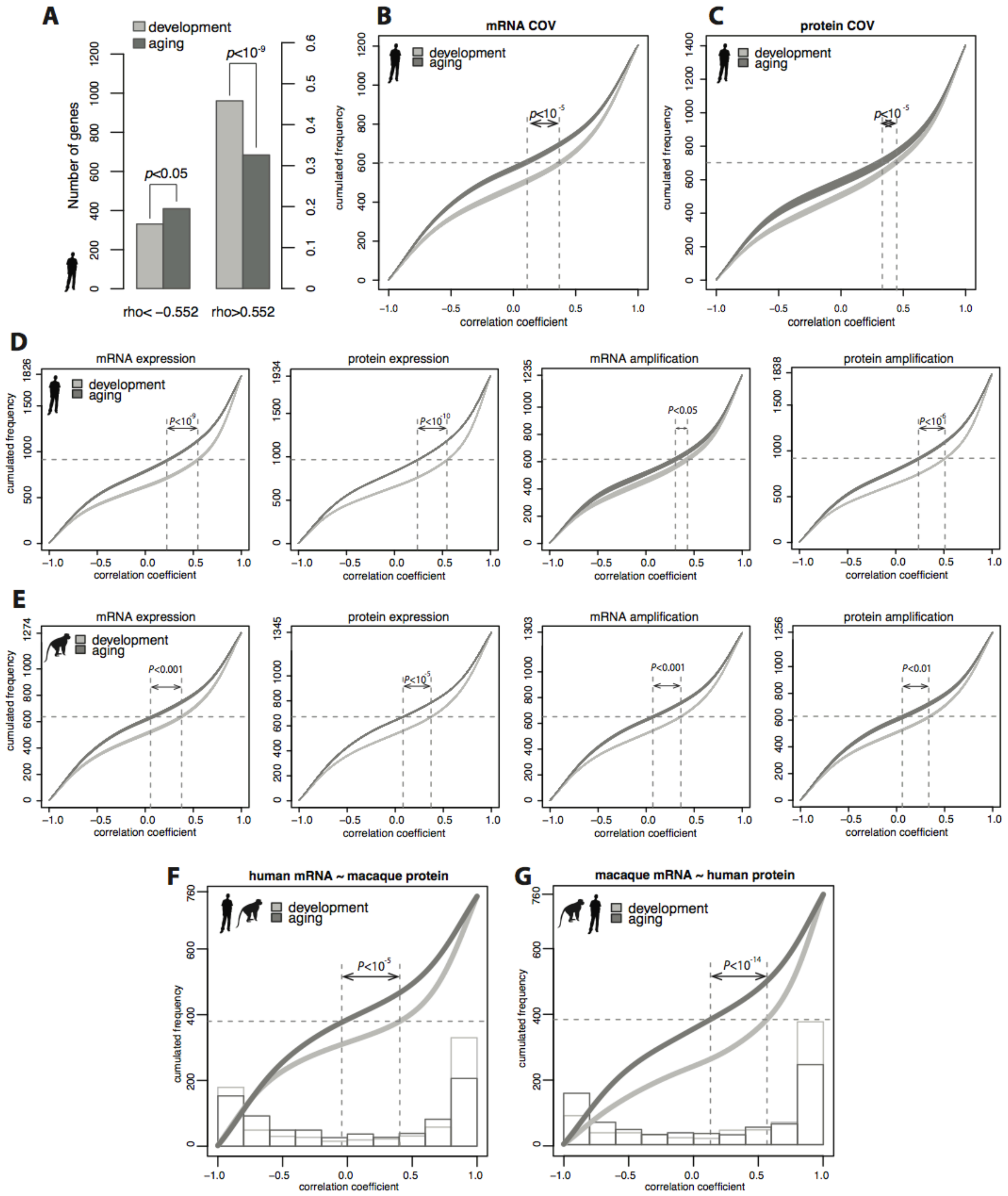


**Figure S4. Robustness and reproducibility of increased decoupling of mRNA and protein expression levels in aging.**

(A) Bar plot shows the numbers of significantly positively/negatively correlated genes identified in the human PFC time-series data in development (light color) and aging (dark color) intervals. The y-axis on the right shows the ratio of gene numbers compared to the total amount of 1,963. *P*-values of chi-square tests are marked for both comparisons.

(B-C) The chance distribution of cumulative frequency of Spearman's rank correlation coefficients based on mRNA (B) and protein (C) expression in developmental (light grey curves) and aging

(dark grey curves) intervals, of human time-series data. Distribution of curves were determined by subsampling 1,000 times using an equalized distribution of coefficient of variation between development and aging intervals. The dashed lines show median values of the curve distributions, the p-values show significance of the difference between medians estimated by Wilcoxon test.

(D-E) The chance distribution of cumulative frequency of Spearman's rank correlation coefficients based on mRNA and protein expression changes in developmental (light grey curves) and aging (dark grey curves) intervals, of human (D) and macaque (E) time-series data. Distribution of curves were made by 1,000 times of subsampling using equalized distribution between development and aging intervals, of mRNA expression, protein expression, mRNA expression change amplitudes, and protein expression change amplitudes. The dashed lines show median values of the curve distributions, the p-values show significance of the difference between medians estimated by Wilcoxon test.

(F-G) The cumulative frequency of Spearman's rank correlation coefficients based on mRNA and protein expression changes in developmental (light grey curves) and aging (dark grey curves) intervals respectively. The dashed lines show median values of the curves, the p-values show significance of the difference between medians estimated by Wilcoxon tests. The histograms at the bottom of the panels show distributions of Spearman's rank correlation coefficients in developmental (light grey) and aging (dark grey) intervals composing the curves. The results are shown for human mRNA with macaque protein (F), and vice versa (G). The y-axis shows numbers of genes used in each comparison.

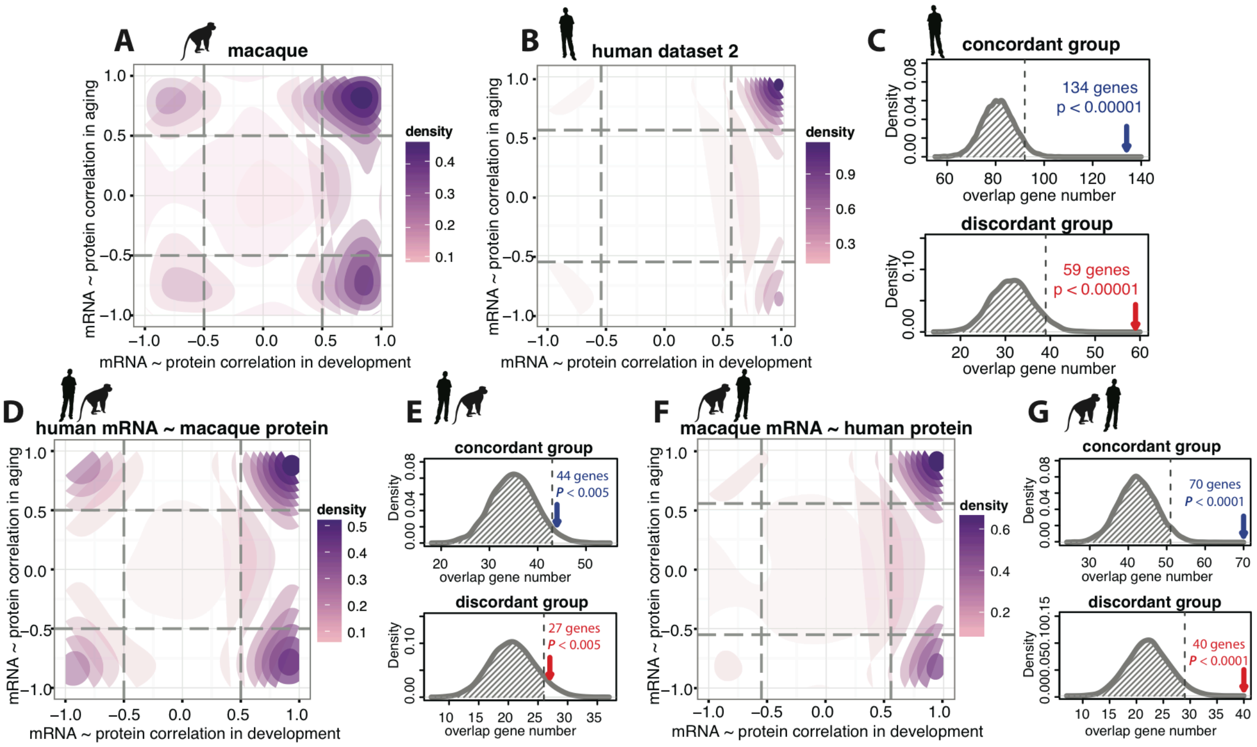


**Figure S5. Robustness and reproducibility of concordant and discordant gene groups.**

Two-dimensional density plot showing distribution of mRNA-protein Spearman's rank correlation coefficients measured during developmental (x-axis) and aging (y-axis) intervals of macaque time-series data (A), of another human RNA-seq time-series dataset (B), of human mRNA with macaque

protein (D) and vice versa (F). The grey dashed lines show the correlation coefficient cut-offs used to define concordant and discordant gene groups ($p < 0.05$, Spearman's rank correlation; FDR < 0.05, permutations).

The overlap of concordant and discordant gene groups between human time-series and another human RNA-seq time-series dataset (C), of human mRNA with macaque protein (E) and vice versa (G). The arrows show numbers of overlap found in actual data; the distributions show chance overlap estimated by 1,000 permutations of gene labels. The dashed line indicates 95% quantile of the distribution.

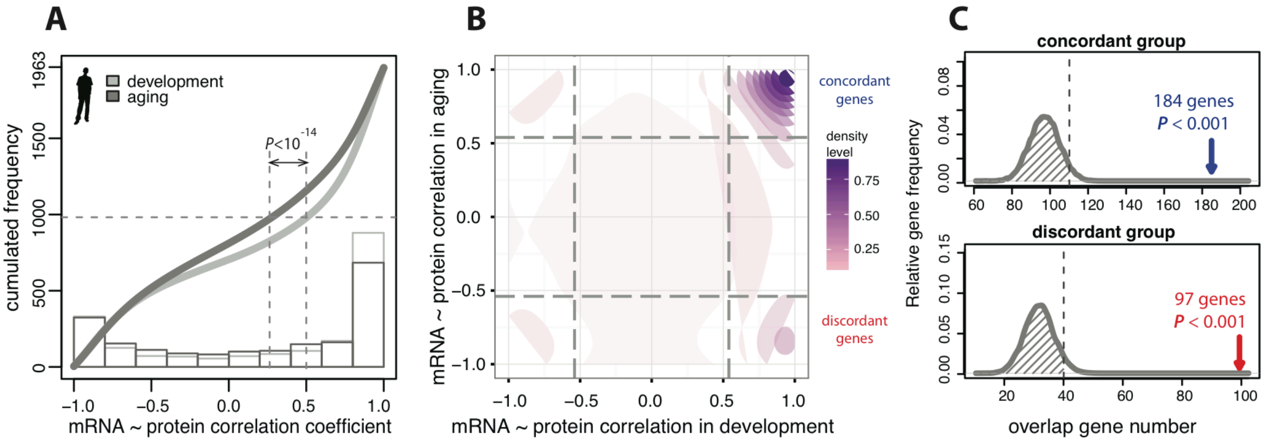


**Figure S6. Relationship of age-dependent mRNA and protein level changes measured in the same set of 10 human tissue samples.**

(A) The cumulative frequency of Spearman's rank correlation coefficients based on mRNA and protein expression changes in developmental (light grey curves) and aging (dark grey curves) intervals respectively. The dashed lines show median values of the curves, the p-values show significance of the difference between medians estimated by Wilcoxon test. The histograms at the bottom of the panels show distributions of Spearman's rank correlation coefficients in developmental (light grey) and aging (dark grey) intervals composing the curves. The y-axis shows numbers of genes used in each comparison.

(B) Two-dimensional density plot showing distribution of mRNA-protein Spearman's rank correlation coefficients measured during developmental (x-axis) and aging (y-axis) intervals in human PFC, including only ten samples matched between mRNA dataset 1 and the protein dataset. The grey dashed lines show the correlation coefficient cut-offs used to define concordant and discordant gene groups ($p < 0.05$, Spearman's rank correlation; FDR < 0.05, permutations).

(C) The overlap of concordant and discordant gene groups between human full list and human time-series including only ten samples matched between mRNA dataset 1 and protein dataset. The arrows show numbers of overlap found in actual data; the distributions show chance overlap estimated by 1,000 permutations of gene labels. The dashed line indicates 95% quantile of the distribution.

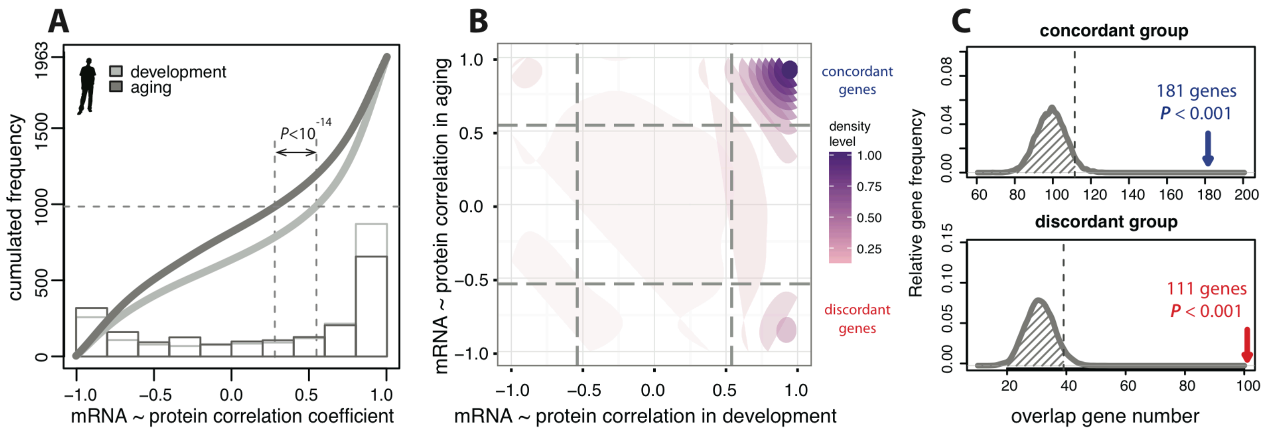


**Figure S7. Relationship of age-dependent mRNA and protein level changes measured in the same nine Caucasian human tissue samples.**

(A) The cumulative frequency of Spearman's rank correlation coefficients based on mRNA and protein expression changes in developmental (light grey curves) and aging (dark grey curves) intervals respectively. The dashed lines show median values of the curves, the p-values show significance of the difference between medians estimated by Wilcoxon test. The histograms at the bottom of the panels show distributions of Spearman's rank correlation coefficients in developmental (light grey) and aging (dark grey) intervals composing the curves. The y-axis shows numbers of genes used in each comparison.

(B) Two-dimensional density plot showing distribution of mRNA-protein Spearman's rank correlation coefficients measured during developmental (x-axis) and aging (y-axis) intervals in human PFC, including only nine Caucasian samples. The grey dashed lines show the correlation coefficient cut-offs used to define concordant and discordant gene groups ($p < 0.05$, Spearman's rank correlation; FDR < 0.05, permutations).

(C) The overlap of concordant and discordant gene groups between human full list and human time-series including only nine Caucasian samples. The arrows show numbers of overlap found in actual data; the distributions show chance overlap estimated by 1,000 permutations of gene labels. The dashed line indicates 95% quantile of the distribution.

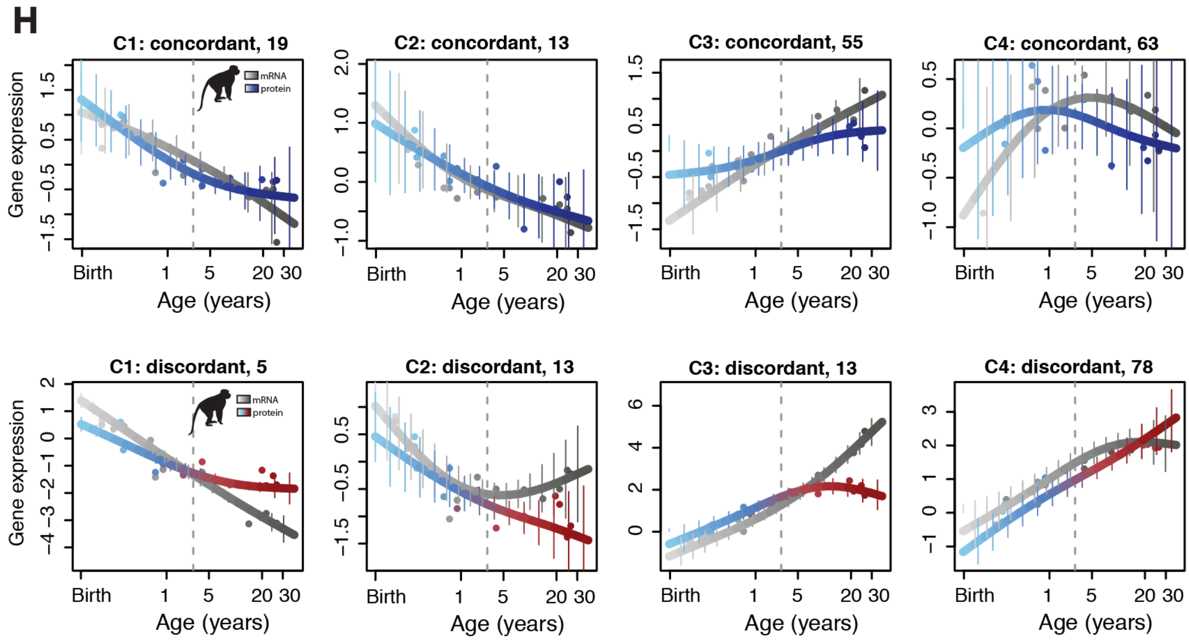


**Figure S8. Reproducibility of concordant and discordant gene clusters in rhesus macaque.**

mRNA/protein expression of macaque time-series data in the four human patterns of concordant (upper panels) and discordant (lower panels) gene groups. The curves show the average mRNA (grey) and protein (colored) expression calculated using cubic spline regression. The points show the mean expression in each individual. The y-axis shows mRNA and protein expression, normalized to the mean and standard deviation of corresponding expression levels in developmental interval. The vertical error bars show the standard deviation range of the curves. The pattern number and the number of genes in each group detected in macaque time-series data are shown on the top of the panels. The vertical dashed line marks separation of developmental and aging intervals.

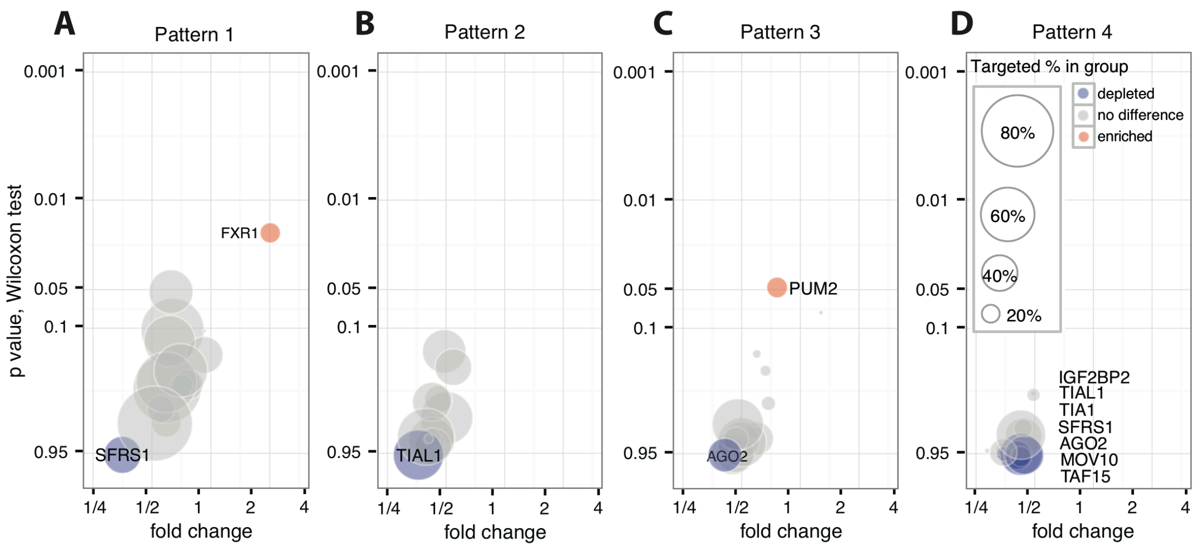

**Figure S9. Reciprocal analysis of common RBP regulator in concordant groups.**

Enrichment of RBP binding sites within concordant genes in each of the four main expression patterns, compared to discordant genes from the same pattern. The x-axis shows the enrichment fold-change based on the binding site number, the y-axis shows significance of binding site density difference. Each circle represents one RBP. The circle radius shows the proportion of concordant genes targeted by the RBP within the group. Colors indicate RBPs showing significant enrichment (red), no difference (grey) or significant depletion (blue) of binding sites within concordant genes compared to discordant ones.

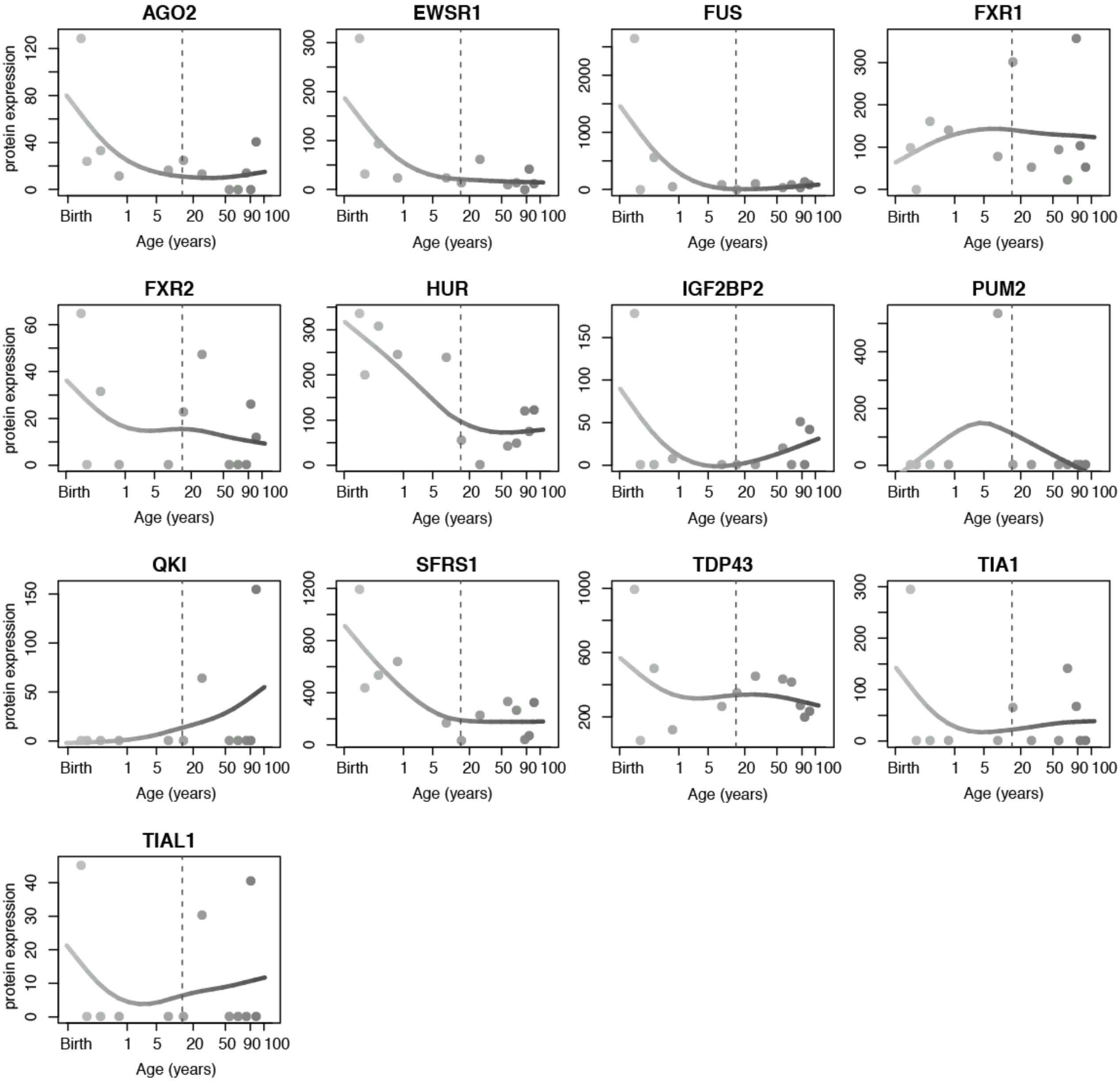


**Figure S10. Protein expression of human RBPs.**

The curves calculated using cubic spline regression and the individual data points show the protein expression levels of 13 RBPs detected in our data of the 17 RBPs with CLIP experimental data used in the analysis. The symbols show the expression in each individual. The y-axis shows the

normalized spectral abundance factor (NSAF) of protein expression. The vertical dashed line marks separation of developmental and aging intervals.

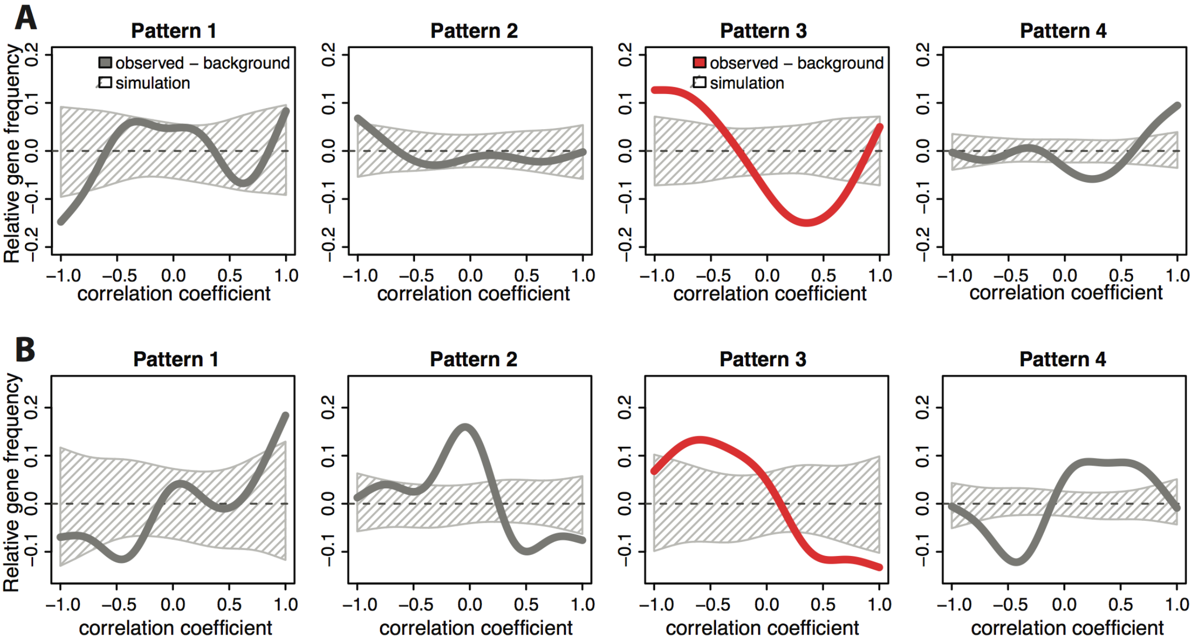


**Figure S11. Robustness of miRNAs' regulation on decoupling protein from mRNA across different target prediction strategies.**

(A-B) Difference between the distribution of predicted miRNA-target correlations and the chance background. The curves show predicted miRNA-target distribution of Spearman's rank correlation coefficients measured based on expression profiles of age-dependent miRNA and protein expression of their predicted target transcripts in aging interval. miRNA target prediction is made by mirDB3.0 (A) and COMETA (B) [1, 2]. The background chance distribution was estimated by generating the same number of predicted miRNA-target pairs based on randomly chosen age-dependent miRNA and target genes within discordant groups of each pattern for 1,000 times. The shaded areas show the 95% confidence intervals of the correlation coefficients' chance distributions. The median of background chance distribution was subtracted from both the predicted miRNA-target and the chance background distributions.

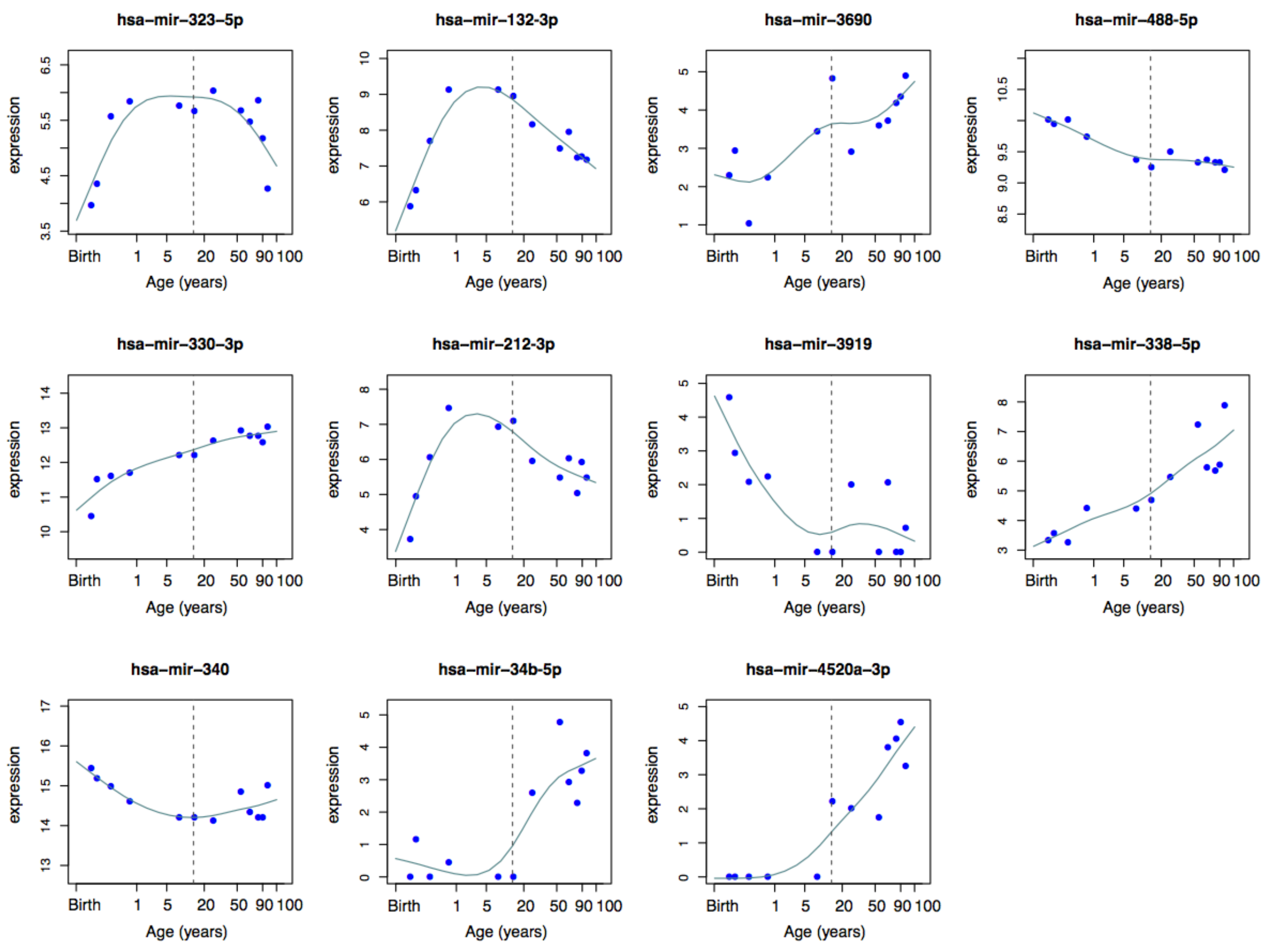


**Figure S12. Expression of 11 age-dependent miRNAs with predicted target enrichment in discordant gene groups.**

The circles show the miRNA expression in each individual. The curves were fitted to the data using cubic spline regression. The y-axis shows miRNA expression calculated as the transcripts per million reads (TPM). The vertical dashed line marks separation of developmental and aging intervals.

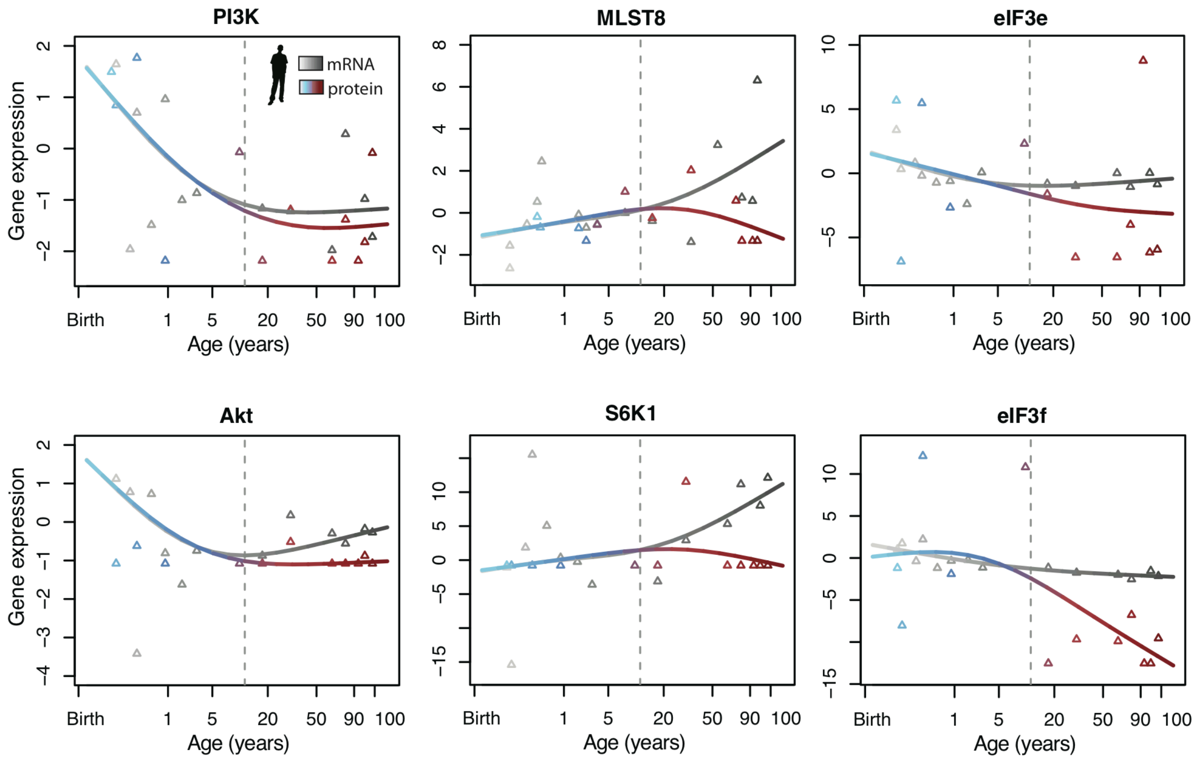


**Figure S13. mRNA/protein expression of discordant genes in PI3K-Akt-mTORC1 signaling cascade.**

The curves show the mRNA (grey) and protein (colored) expression of the six discordant genes in the PI3K-Akt_mTORC1 signaling cascade, calculated using cubic spline regression. The symbols show the expression in each individual. The y-axis shows mRNA and protein expression, normalized to the mean and standard deviation of corresponding expression levels in developmental interval. The vertical dashed line marks separation of developmental and aging intervals.

# Supplemental Tables

## Table S1. Dataset information

| Species | Data classification | No. of datasets | Status of dataset |
|---|---|---|---|
| *Homo sapiens* | mRNA sequencing | 2 | Downloaded from SRP005169 |
| | | | Downloaded from GSE51264 |
| | miRNA sequencing | 1 | Downloaded from GSE18069 |
| | Protein label-free quantitative mass spectrometry | 1 | Uploaded to PASS00505 |
| *Macaca* | mRNA sequencing | 1 | Downloaded from SRP005169 |

| | | | |
|---|---|---|---|
| *mulatta* | Protein label-free quantitative mass spectrometry | 1 | Uploaded to PASS00505 |

**Table S2. Sample information for downloaded human mRNA dataset 1.**

| Brain Bank ID | Age | | Sex | Source | Ethnicity |
|---|---|---|---|---|---|
| | Years | Days | | | |
| Maryl_447 | 0 | 2 | M | NICHD[1] | Caucasian |
| Maryl_779 | 0 | 4 | M | NICHD | African American |
| Maryl_1157 | 0 | 19 | F | NICHD | Caucasian |
| Maryl_759 | 0 | 34 | M | NICHD | Caucasian |
| Maryl_1055 | 0 | 94 | M | NICHD | Caucasian |
| Maryl_1281 | 0 | 204 | M | NICHD | African American |
| 1453 | 1 | 78 | M | NICHD | African American |
| 1275 | 2 | 57 | F | NICHD | African American |
| 1908 | 13 | 360 | M | NICHD | Caucasian |
| 605 | 25 | 152 | M | NICHD | African American |
| 1578 | 53 | 112 | M | NICHD | Caucasian |
| S06/117 300 GFS1 | 66 | 0 | M | NBB[2] | Caucasian |
| S01/118 300 GFS1 | 88 | 0 | M | NBB | Caucasian |
| S00/047 100 B1 | 98 | 0 | M | NBB | Caucasian |

shared with protein dataset | shared with mRNA dataset 2 | shared with both protein dataset and mRNA dataset 2

[1]NICHD: Brain and Tissue Bank for Developmental Disorders at the University of Maryland, USA.

[2]NBB: Netherlands Brain Bank, Amsterdam, Netherlands.

**Table S3. Sample information for downloaded rhesus mRNA dataset.**

| Brain Bank ID | Age | | Sex |
|---|---|---|---|
| | Years | Days | |
| NB0902 | 0 | 1 | M |
| NB0901 | 0 | 1 | M |
| NB0905 | 0 | 7 | M |
| 0705 | 0 | 16 | M |
| 0703 | 0 | 22 | M |
| 070141 | 0 | 153 | M |
| 070133 | 0 | 207 | M |
| 06711 | 0 | 310 | M |
| 051095 | 2 | 9 | M |

| | | | |
|---|---|---|---|
| 03071 | 4 | 27 | M |
| 98073 | 9 | 104 | M |
| 92107 | 15 | 3 | M |
| 85091 | 22 | 74 | M |
| 198100 | 26 | 28 | M |
| QDL II | 28 | 0 | F |

shared with protein dataset

## Table S4. Number of reads in mRNA dataset 1 under our mapping procedure.

| Species | Brain Bank ID | Total reads | Non-PCR duplicated reads | Mapped Reads | Uniquely mapped reads |
|---|---|---|---|---|---|
| *Homo sapiens* | Maryl_447 | 21,142,703 | 18,673,872 | 12,784,641 | 9,877,087 |
| | Maryl_779 | 21,284,713 | 15,645,640 | 13,192,176 | 10,366,368 |
| | Maryl_1157 | 20,597,598 | 15,841,854 | 10,751,463 | 8,188,203 |
| | Maryl_759 | 23,512,147 | 15,922,713 | 14,868,856 | 12,092,512 |
| | Maryl_1055 | 23,239,386 | 16,455,012 | 13,708,226 | 10,227,074 |
| | Maryl_1281 | 22,488,710 | 15,624,318 | 13,314,075 | 10,378,509 |
| | 1453 | 23,866,146 | 15,212,377 | 14,854,062 | 11,907,830 |
| | 1275 | 17,683,503 | 11,810,470 | 10,488,454 | 8,188,883 |
| | 1908 | 19,757,403 | 11,950,952 | 11,233,343 | 9,077,002 |
| | 605 | 23,121,948 | 15,591,218 | 13,912,559 | 11,335,574 |
| | 1578 | 15,897,722 | 10,335,192 | 9,367,250 | 7,398,830 |
| | S06/117 300 GFS1 | 20,880,231 | 14,013,080 | 12,987,349 | 10,277,496 |
| | S01/118 300 GFS1 | 20,927,309 | 13,876,966 | 12,897,849 | 9,933,448 |
| | S00/047 100 B1 | 20,185,086 | 13,667,334 | 12,898,325 | 9,886,290 |
| *Macaca mulatta* | NB0902 | 35,647,306 | 25,669,432 | 23,416,604 | 17,407,482 |
| | NB0901 | 36,207,576 | 26,563,378 | 21,439,014 | 16,342,374 |
| | NB0905 | 35,368,530 | 24,532,147 | 21,297,102 | 16,079,832 |
| | 0705 | 33,944,540 | 22,406,289 | 20,908,734 | 15,857,100 |
| | 0703 | 35,150,179 | 22,230,204 | 20,449,921 | 15,603,915 |
| | 070141 | 36,074,539 | 22,447,410 | 21,090,285 | 15,961,829 |
| | 070133 | 34,525,819 | 24,739,753 | 21,345,050 | 16,201,243 |
| | 06711 | 31,005,239 | 21,383,306 | 16,898,212 | 14,672,893 |
| | 051095 | 32,143,377 | 23,989,987 | 16,176,015 | 13,052,900 |
| | 03071 | 30,998,685 | 22,908,107 | 15,946,795 | 12,463,526 |
| | 98073 | 32,106,698 | 22,293,434 | 16,460,124 | 12,463,526 |

| | | | | | |
|---|---|---|---|---|---|
| | 92107 | 31,450,483 | 23,045,855 | 16,579,295 | 12,682,847 |
| | 85091 | 28,942,443 | 23,254,017 | 15,316,963 | 12,564,802 |
| | 198100 | 29,315,091 | 21,470,124 | 14,685,960 | 12,050,290 |
| | QDL II | 29,042,277 | 20,971,569 | 14,425,718 | 11,599,236 |

**Table S5. Sample information for human protein dataset and downloaded miRNA RNA-sequencing dataset.**

| Brain Bank ID | Age | | Sex | Source | Ethnicity |
|---|---|---|---|---|---|
| | Years | Days | | | |
| Maryl_447 | 0 | 2 | M | NICHD[1] | Caucasian |
| Maryl_779 | 0 | 4 | M | NICHD | African American |
| Maryl_759 | 0 | 34 | M | NICHD | Caucasian |
| Maryl_1281 | 0 | 204 | M | NICHD | African American |
| 1860 | 8 | 2 | M | NICHD | Caucasian |
| 1908 | 13 | 360 | M | NICHD | Caucasian |
| 605 | 25 | 152 | M | NICHD | African American |
| 1578 | 53 | 112 | M | NICHD | Caucasian |
| S06/117 300 GFS1 | 66 | 0 | M | NBB[2] | Caucasian |
| S04/057 100 B1 | 80 | 0 | M | NBB | Caucasian |
| S01/118 300 GFS1 | 88 | 0 | M | NBB | Caucasian |
| S00/047 100 B1 | 98 | 0 | M | NBB | Caucasian |

shared with mRNA dataset 1 — shared with mRNA dataset 2 — shared with both mRNA dataset 1 and dataset 2

All samples were shared by the human protein dataset measured in this study and downloaded miRNA RNA-seq dataset.

[1]NICHD: Brain and Tissue Bank for Developmental Disorders at the University of Maryland, USA.

[2]NBB: Netherlands Brain Bank, Amsterdam, Netherlands.

**Table S6. Sample information for rhesus protein dataset**

| Brain Bank ID | Age | | Sex |
|---|---|---|---|
| | Years | Days | |
| 0705 | 0 | 16 | M |
| 0704 | 0 | 20 | M |
| 070141 | 0 | 153 | M |
| 070133 | 0 | 207 | M |

| | | | |
|---|---|---|---|
| 06711 | 0 | 310 | M |
| 051095 | 2 | 9 | M |
| 03071 | 4 | 27 | M |
| 98073 | 9 | 104 | M |
| 87015 | 20 | 91 | M |
| 85091 | 22 | 74 | M |
| 198100 | 26 | 28 | M |
| QDL II | 28 | 0 | F |

shared with mRNA dataset 1

## Table S7. Number of detected spectra and identified peptides.

| Species | Brain Bank ID | Total Spectra | Peptide number (FDR 1%) |
|---|---|---|---|
| *Homo sapiens* | Maryl_447 | 186,923 | 58,886 |
| | Maryl_779 | 175,682 | 44,742 |
| | Maryl_759 | 196,469 | 67,277 |
| | Maryl_1281 | 205,694 | 71,542 |
| | 1860 | 186,838 | 57,407 |
| | 1908 | 198,458 | 66,173 |
| | 605 | 190,676 | 62,316 |
| | 1578 | 199,656 | 68,090 |
| | S06/117 300 GFS1 | 196,574 | 63,911 |
| | S04/057 100 B1 | 196,979 | 64,322 |
| | S01/118 300 GFS1 | 192,780 | 64,145 |
| | S00/047 100 B1 | 193,960 | 65,447 |
| *Macaca mulatta* | 0705 | 183,296 | 57,432 |
| | 0704 | 184,174 | 60,203 |
| | 070141 | 184,364 | 60,727 |
| | 070133 | 182,599 | 53,906 |
| | 06711 | 177,009 | 49,228 |
| | 051095 | 179,522 | 57,125 |
| | 03071 | 198,533 | 62,514 |
| | 98073 | 184,055 | 57,201 |
| | 87015 | 186,218 | 61,082 |
| | 85091 | 184,524 | 56,337 |
| | 198100 | 183,549 | 55,074 |

| | | | |
|---|---|---|---|
| | QDL II | 190,240 | 59,051 |

**Table S8. Sample information for downloaded human mRNA dataset 2.**

| Brain Bank ID | Age | | Sex | Source | Ethnicity |
|---|---|---|---|---|---|
| | Years | Days | | | |
| Maryl_447 | 0 | 2 | M | NICHD[1] | Caucasian |
| Maryl_779 | 0 | 4 | M | NICHD | African American |
| Maryl_398 | 0 | 16 | F | NICHD | African American |
| Maryl_1157 | 0 | 19 | F | NICHD | Caucasian |
| Maryl_759 | 0 | 34 | M | NICHD | Caucasian |
| Maryl_1055 | 0 | 94 | M | NICHD | Caucasian |
| 5183 | 0 | 107 | M | NICHD | Caucasian |
| 1303 | 0 | 212 | M | NICHD | Caucasian |
| 1453 | 1 | 78 | M | NICHD | African American |
| 814 | 1 | 123 | M | NICHD | Caucasian |
| 1275 | 2 | 57 | F | NICHD | African American |
| 1791 | 2 | 286 | F | NICHD | African American |
| 6736 | 4 | 0 | F | NICHD | Caucasian |
| 1185 | 4 | 258 | M | NICHD | Caucasian |
| 4907 | 4 | 274 | F | NICHD | African American |
| 4898 | 7 | 272 | M | NICHD | Caucasian |
| 1860 | 8 | 2 | M | NICHD | Caucasian |
| 1706 | 8 | 214 | F | NICHD | African American |
| 5161 | 10 | 262 | F | NICHD | African American |
| M3228 | 11 | 294 | M | NICHD | Caucasian |
| 1908 | 13 | 360 | M | NICHD | Caucasian |
| 5242 | 15 | 119 | M | NICHD | Caucasian |
| 7387 | 17 | 0 | M | NICHD | Caucasian |
| 5251 | 19 | 0 | M | NICHD | Caucasian |
| 4548 | 20 | 63 | F | NICHD | African American |
| 1846 | 20 | 221 | F | NICHD | Caucasian |
| 1442 | 22 | 322 | M | NICHD | Caucasian |
| 7738 | 24 | 0 | M | HBTRC[2] | African American |
| 602 | 27 | 42 | M | NICHD | African American |
| B-24 | 28 | 0 | F | HBTRC | Caucasian |
| 1502 | 29 | 363 | M | NICHD | Caucasian |
| 7344 | 36 | 0 | F | NICHD | Caucasian |

| | | | | | |
|---|---|---|---|---|---|
| 7561 | 39 | 0 | M | HBTRC | Caucasian |
| 1134 | 41 | 241 | M | NICHD | Caucasian |
| 6259 | 50 | 0 | M | HBTRC | Caucasian |
| 1578 | 53 | 112 | M | NICHD | Caucasian |
| 6860 | 56 | 0 | M | HBTRC | Caucasian |
| 4263 | 61 | 187 | M | NICHD | Caucasian |

shared with protein dataset   shared with mRNA dataset 1   shared with both protein dataset and mRNA dataset 1

[1]NICHD: Brain and Tissue Bank for Developmental Disorders at the University of Maryland, USA.

[2]HBTRC: Harvard Brain Tissue Resource Center.

**Table S10. Data sets of CLIP experiments used in the analysis.**

All data sets of CLIP experiments (Hits-CLIP, Par-CLIP and iCLIP) used in the analysis were collated from the published literature. Precise positional target site information was extracted from the NCBI Gene Expression Omnibus (http://www.ncbi.nlm.nih.gov/geo), from the Sequence Reads Archive (http://www.ncbi.nlm.nih.gov/sra), from the EMBL-EBI ArrayExpress (http://www.ebi.ac.uk/arrayexpress/) with the accession ID beginning with 'GSE', 'SRA' and 'E-M' respectively, or directly from the supplementary data of the literature that did not include accession ID.

| RBP name | Experiment | Cell line | Reference | Accession ID |
|---|---|---|---|---|
| AGO2 | Hits-CLIP | HEK293 | [3] | GSE28865 |
| EWSR1<br>FUS<br>TAF15 | Par-CLIP | HEK293 | [4] | SRA025082 |
| FMR1<br>FXR1<br>FXR2 | Par-CLIP | HEK293 | [5] | GSE39682 |
| hnRNPC | iCLIP | Hela | [6] | -- |
| HuR | Hits-CLIP | HEK293 | [3] | GSE28865 |
| IGF2BP2 | Par-CLIP | HEK293 | [7] | GSE21918 |
| MOV10 | Par-CLIP | HEK293 | [8] | GSE37524 |
| PUM2<br>QKI | Par-CLIP<br>Par-CLIP | HEK293<br>HEK293 | [7] | GSE21918 |
| SFRS1 | Hits-CLIP | HEK293 | [9] | -- |
| TDP43 | iCLIP | neuroblastoma | [10] | E-MTAB-530 |
| TIA1<br>TIAL1 | iCLIP | Hela | [11] | -- |

**Table S11. Age-dependent miRNAs with enriched predicted target number in discordant group of four patterns.**

| Pattern | Enriched miRNA number | Enriched miRNAs | Predicted target gene number | Predicted target genes |
|---|---|---|---|---|
| 1 | 3 | hsa-mir-323-5p | 2 | CHD3, MDGA2 |
| | | hsa-mir-330-3p | 5 | EFNB2, MTMR9, NASP, TENM3, UBA6 |
| | | hsa-mir-340 | 6 | EFNB2, MDGA2, MTMR9, PHF14, RALA, TENM3 |
| 2 | 0 | -- | -- | -- |
| 3 | 7 | hsa-mir-132-3p | 2 | CC2D1B, SLC2A1 |
| | | hsa-mir-212-3p | 2 | CC2D1B, SLC2A1 |
| | | hsa-mir-34b-5p | 2 | ARHGAP1, FAM171A1 |
| | | hsa-mir-3690 | 2 | AHCYL1, DIP2B |
| | | hsa-mir-3919 | 4 | ARHGAP1, GOT2, RTN4IP1, SYNJ1 |
| | | hsa-mir-4520a-3p | 2 | ARHGAP1, MTCH1 |
| | | hsa-mir-488-5p | 2 | AP3B1, SLC2A1 |
| 4 | 2 | hsa-mir-338-5p | 11 | AAK1, AKAP12, CLTC, HPRT1, LOC101930324, NSF, PCDH7, PJA2, RAB3A, SYT1, TPD52 |
| | | hsa-mir-488-5p | 7 | AAK1, ATP1A1, ATP1B1, NAPB, PCBH7, TPD52, YWHAB |

# Supplemental Experimental Procedures

Analyses were performed in the R environment (http://www. r-project.org/). We used WikiGene Name obtained from Ensembl (v. 73; http://www.ensembl.org). All miRNA names are based on miRBase (v. 17; http:// www.mirbase.org/).

## Ethics Statement

Informed consent for the use of human tissues for research was obtained in writing from all donors or their next of kin. All macaques used in this study suffered sudden deaths for reasons other than their participation in this study and without any relation to the tissue used. The Biomedical Research Ethics Committee of Shanghai Institutes for Biological Sciences completed the review of the use and care of animals in this research project (approval ID: ER-SIBS-260802P).

## Sample collection

Healthy human tissues were obtained from the NICHD Brain and Tissue Bank for Developmental Disorders at the University of Maryland, USA and the Netherlands Brain Bank. Macaque tissues were obtained from the Suzhou Experimental Animal Centre in China.

The role of the NICHD Brain and Tissue Bank and the Netherlands Brain Bank are to distribute tissue and, therefore, cannot endorse the studies performed or the interpretation of results. All human subjects were defined as normal controls by forensic pathologists at the NICHD Brain and Tissue Bank and the Netherlands Brain Bank. No subjects who suffered a prolonged agonal state were included.

PFC dissections were made from the frontal part of the superior frontal gyrus, a cortical region approximately corresponding to Brodmann area 9. All samples contained an ~2:1 grey matter to white matter volume ratio.

### Human-macaque consensus reference genome construction

Chained and netted alignment files for human (hg19) and macaque (rheMac3), aligned using BLASTZ [12], were downloaded from the UCSC Genome Browser. On the basis of these alignments, we constructed a human-macaque consensus genome. Specifically, we used the human genome as a reference for all discordant genomic sites, and replaced insertions and deletions with "N"s in the consensus genome. Furthermore, 6 bp regions flanking each insertion or deletion site were masked by "N"s in the resulting human-macaque consensus reference genome.

### Computational pre-processing of mRNA deep sequencing

The deep sequencing data of human and rhesus superior frontal gyrus of the prefrontal cortex (PFC) were obtained from the Sequence Reads Archive (http://www.ncbi.nlm.nih.gov/sra) under the accessions SRP005169 for [13] (Table S1-S3).

To quantify and compare gene expression in the two species in an unbiased manner, we mapped human and rhesus RNA-seq reads to the human-macaque consensus reference genome, using STAR (v. 2.3.0e; [14]) with parameters allowing a maximum of three mismatches and a minimum 90% percentage of non-'N' matches. Potential PCR duplicates were removed by samtools rmdup (v. 0.1.19; [15]). For each mapping procedure, only uniquely mapped reads were used in further analyses.

Gene expression was quantified as the number of reads per kilo-base per million of total mapped reads (RPKM) by GENCODE annotation (v.17; http://www.gencodegenes.org) [16]. The number of reads that map to each gene was counted by Python package HTSeq (v. 0.5.4p2; http://www-huber.embl.de/users/anders/HTSeq/), adding the only parameter of whether the data was from a strand-specific assay. The gene length was defined as the union of all its exons.

Only genes with RPKM $\geqslant$ 1 in more than 2/3 of the samples in one species (human or macaque) were classified as reliably expressed in that species and were used in subsequent analyses.

### Protein sample preparation and Label-Free 2D-MS/MS Thermo-LTQ Proteomics Methods

For extraction, we used 100mg frozen prefrontal cortex samples from 12 human and 12 macaque individuals (Table S5-S6). The 12 human and 12 macaque samples were processed in two batches with 6 individuals in each, with similar age distributions in both batches. Tissue samples were minced, washed in ice-cold PBS and homogenized in ice-cold lysis buffer (8 M urea, 4% CHAPS, 65 mM DTT, 40 mM Tris, cocktail protease inhibitor, 100 mg of tissue/1 ml) using an electric

homogenizer. The resulting protein solutions were sonicated on ice for a total of 3 minutes and then centrifuged at 25,000g for 1 hour at 4°C to remove DNA, RNA and other cell debris. Next, the protein supernatants were precipitated using 5× volumes of precipitation solution (ethanol: acetone: acetic acid = 50:50:0.1, volume ratio) at 4°C overnight, centrifuged and re-suspended in denaturing buffer [6 M guanidine hydrochloride, 100mM Tris, cocktail protease inhibitor, phosphatase inhibitors (1mM sodium orthovanadate and 1mM sodium fluoride), pH 8.3]. Protein concentration was determined using the Bradford assay. Next, 600 μg of protein from each sample was reduced with DTT (100 μg / 1 μl 1M DTT), alkylated with IAA (100 μg / 2 μl 1M IAA), and precipitated again at 4° C overnight (as described above). After centrifugation, the resulting precipitates were re-suspended in digestion buffer (100mM ammonium bicarbonate) and incubated with Trypsin (enzyme:protein = 1:40, mass ratio) at 37°C for 20 hours, followed by ultrafiltration and lyophilization.

Each peptide sample was re-suspended in 50 μl SCX loading buffer, loaded on a SCX (Strong Cation Exchange) column (Column Technology Inc., CA, USA) and eluted using a pH continuous gradient buffer (from pH 2.5-8.5), resulting in 10 fractions. Each of these fractions was then automatically loaded on one of two RP (Reversed Phase) alternative trap columns, by switching to the other RP column every 3 hours. Analysis was performed on the LTQ mass spectrometer equipped with a metal needle electrospray interface mass spectrometer (ThermoFinnigan, San Jose, CA, USA) in a data-dependent collection model (each full scan followed by ten MS/MS scans of most intense ions).

**Computational pre-processing of quantitative proteomics**

Peptides were identified by searching spectrums against the UniProt Knowledgebase (UniProKB) complete proteome human set [17], using the database search engine MS-GF+ [18] with default modification parameters. Each peptide had to be fully tryptic, and had a length of at least 6 amino acids. The parent mass tolerance was set to 2.5 Da. We estimated peptide-spectrum matches (PSM) level FDR of peptide identification by searching the combined database of target dataset and the reversed decoy database. Only peptides with FDR<1% were considered. For each database searching procedure, only spectrums matched to unique peptide were used in further analyses. Ten MS/MS scans of each sample were combined together.

Peptide data were mapped per gene to Ensembl genes by UniProtKB/Swiss-Prot and UniProtKB/TrEMBL annotations. Criteria for protein identification included detection of at least 2 unique peptides. Ambiguous peptides and redundant proteins were removed.

Quantitation of protein expression of each gene was achieved by the normalized spectral abundance factor (NSAF) [19] to allow the comparison of abundance of individual proteins in multiple independent samples. The count of MS/MS spectra assigned to a protein was normalized to the length of the protein (total number of amino acids), resulting in a Spectral Abundance Factor (SAF). Each SAF was further normalized against the sum of all SAFs in one sample, resulting in the NSAF value.

Only genes with mean NSAF≥1 and a positive NSAF value in at least 6 of the 12 individuals were classified as reliably detected in a species (human or macaque), and were included in the downstream analysis.

**Age-scales for modeling expression change.**

A $log_2$-age scale is suited for modeling exponential changes during early development (e.g. [20]) while linear age allows better identification of linear changes that occur during aging [21, 22]. To avoid bias toward either age stage, we used the power of 0.25 of donor age to simultaneously capture developmental and aging-dependent changes. Using the power of 0.25 of ages also ensures more uniform distribution of samples across age scale, compared to the other two scales. At the same time, it limits heteroscedasticity across ages, which pertains to the assumption of parametric regression models that errors be normally distributed and homogeneous among samples. We therefore used 0.25-power-based estimates when testing for age-effects. The choice of age scale does not qualitatively affect our results regarding mRNA-protein decoupling.

## Computational pre-processing of age-dependent genes

We followed the steps of [23] to test the effect of age on mRNA expression level, using polynomial regression models. We used the power of 0.25 of donor age to simultaneously capture developmental and aging-dependent changes (as mentioned above). For each detected gene, we choose the best regression model with scaled age as predictor and mRNA expression level as response, using families of polynomial regression models and the "adjusted $r^2$" criterion. The most complex of these models is defined in the formula:

$$Y_{ij} = \beta_{0i} + \beta_{1i} A_j + \beta_{2i} A_j^2 + \beta_{3i} A_j^3 + \varepsilon_{ij},$$

where $Y_{ij}$ is the expression level for gene i and subject j, $A_j$ is the age of the subject j, and $\varepsilon_{ij}$ is the error term. The sum of squared errors in this model are compared to the null model,

$$Y_{ij} = \beta_{0i} + \varepsilon_{ij}.$$

Alternative models are the above models' subsets, including linear and quadratic ones.

The significance of the chosen regression model was estimated using the F-test, and Benjamini-Hochberg correction was carried out for all tested genes as multiple test correction.

We used the power of 0.25 of donor age (as mentioned above) and fitted spline curves to the expression data to describe change with age (degree of freedom=4).

We corrected for life-history differences between humans and macaques following the formula based on a number of resources [24, 25]: $age_{human} = 2.257 + 2.953*age_{macaque}$ to transform macaque age into the same scale as human. We used the power of 0.25 of re-scaled macaque ages and fitted spline curves to the expression data to describe change with age (degree of freedom=4) in all downstream analysis.

## Analysis of mRNA-protein disparity

To analyze the correlation between transcriptome and proteomics in the classical framework of human lifespan, we separated "development" interval (postnatal life interval prior to sexual maturity) and "aging" interval (life interval posterior to sexual maturity), with 14 years as borderline following the commonly-used age intervals for pre- and post-adulthood [21, 26]. For both periods we used data from similar numbers of individuals. About 4 years was used as borderline for macaques based on the age formula mentioned above.

While our samples' ages are not uniform along the age-range, and the mRNA and protein datasets have different age windows, we interpolated 10 uniformed points along the age range of each period, at the 0.25-powered age scale (see also Figure S2).

Spearman's rank correlation coefficient *rho* was calculated for each gene for development and aging periods respectively, between mRNA and protein expression levels, based on interpolated points of spline curves.

We carried out a simulation 10,000 times, with a randomized subset of the original data under the same scale and distribution. We calculated ±0.552, as the 95% cut-off points of Spearman's rank correlation coefficient two-tailed distribution by simulation, which are used as cut-offs with *FDR* < 5% of significant correlation coefficient in the downstream analysis.

**Equalization of variation, expression and amplitude**

To preclude the effects led by different variation (standard deviation (sd) and coefficient of variation (cov)), by different expression levels during development and aging life-intervals, or by higher absolute expression changing rate within development, expression profiles were equalized by sd and cov, by average expression levels, as well as equalization of gene expression change amplitudes, between developmental and aging intervals.

We simulated the distribution of the common part of the expression/amplitude curves of developmental and aging intervals. Then, according to this distribution, we subsampled 1,000 times from age-dependent genes, for each species. In each subsampling, we compared the median of correlation distributions between developmental and aging intervals by Wilcoxon test.

**Overlap of concordant/discordant gene groups between databases**

We estimated the reproducibility of concordant and discordant gene groups resulted from original human time-series dataset, with gene groups resulted independently from (1) macaque time-series dataset, (2) a different human RNA-seq time-series dataset, (3) human mRNA and macaque protein expression, and (4) vice versa. We subsampled 1,000 times from all the age-dependent genes, of two datasets. In each subsampling, we selected genes from each dataset of the same number as concordant/discordant gene groups and checked the number of overlapped genes, and compared real overlap amount with simulated overlap distribution.

**Clustering genes into groups**

We grouped concordant and discordant genes into 4 patterns of mRNA expression using k-means clustering. Before clustering, each gene was standardized to mean=0 and standard deviation=1. Because k-means is a heuristic algorithm, we repeated the procedure 1,000 times to determine the most frequent constellation and used this clustering in downstream analysis.

**Functional analysis**

We used the Gene Ontology (GO) categories of biological process (BP), molecular function (MF) and cellular component (CC), together with Kyoto Encyclopedia of Genes and Genomes (KEGG) pathway databases [27, 28] for testing functional enrichment of gene groups. To identify over-representation, we used GeneCoDis3, a non-redundant and modular enrichment analysis tool for functional annotation of gene sets [29] to investigate the putative functions of concordant and discordant gene groups in each of four patterns. Validated detected age-dependent genes were taken as background, and Hypergeometric test *p*-values were adjusted for multiple testing by the Benjamini-Hochberg correction.

## Extraction of exonic RBP binding sites

All data sets of CLIP experiments (including Hits-CLIP, Par-CLIP and iCLIP) used in the analysis were collected from published literatures. Precise positional target site information were extracted from the NCBI Gene Expression Omnibus (http://www.ncbi.nlm.nih.gov/geo), from the Sequence Reads Archive (http://www.ncbi.nlm.nih.gov/sra), from the EMBL-EBI ArrayExpress (http://www.ebi.ac.uk/arrayexpress/), or directly from the supplementary data of those literature without accession IDs (Table S10).

The precise positional RBP target sites extracted from the CLIP data sets were mapped to gene locations by Ensembl gene annotation. The versions of Ensembl annotation were selected corresponding to the versions of the human genome used by the original publications.

We followed the cut-off used in the original literature for each CLIP experiment. Only binding sites passing the cut-off were used in subsequent analyses. To focus on post-transcriptional regulations corresponding to the mature mRNA, we excluded the binding sites located in intronic regions or in intron/exon splicing junctions.

## Key regulator RBP identification

To define a RBP as a key regulator of the disparity gene group of a pattern, we checked three conditions: (1) the regulator should target (has at least one validated binding site on the gene) a significantly higher percentage of discordant genes, compared to the concordant genes in the same pattern. The significance is indicated by $p < 0.05$ in a binomial test after Bonferroni correction between percentages of targeted genes in concordant and discordant gene groups. (2) The regulator should have significantly more binding sites on the exon union of discordant genes compared to the concordant genes in the same pattern. The binding site number enrichment is classified with $p < 0.05$ of one-sided Wilcoxon test after Bonferroni correction between distributions of binding site number on genes in concordant and discordant gene groups. (3) After correction by the length of exon union for each gene, the regulator should have significantly higher density of binding sites in discordant genes compared to the concordant genes in the same pattern. The significance is indicated by the $p < 0.05$ of one-sided Wilcoxon test after Bonferroni correction between distributions of binding site density of genes in concordant and discordant gene groups. A regulator is classified as a key regulator if it fulfills condition (1) and at least one of conditions (2) and (3).

## Computational pre-processing of miRNA deep sequencing

The miRNA deep sequencing data of human superior frontal gyrus of the prefrontal cortex (PFC) were obtained from the NCBI Gene Expression Omnibus (http://www.ncbi.nlm.nih.gov/geo) under series accession no. GSE18069 for [30] (Table S1 and S5).

We followed the procedures of small RNA sequencing data pre-processing and miRNA expression quantification from [31]. In brief, for the small RNA sequencing data pre-processing procedure, all unique sequences were trimmed using the custom trimming procedure, to remove the adapter sequence at the 3'-end. Trimmed sequences (18–28-nt) were mapped to the human genome (hg19) by Bowtie algorithm [32], requiring a perfect match. For the miRNA expression quantification procedure, we downloaded human miRNA annotations, including the sequences and genomic locations of miRNA precursors and mature miRNA sequences, from miRBase version 17 (ref). All perfectly-mapped sequences were used for the following quantification

procedure. First, all sequences mapping within three nucleotides upstream or downstream of the annotated 5'-position of the mature miRNAs were retained. Then, for each mature miRNA, the sequence with a maximal copy number was designated as the reference sequence. Finally, the expression level of each miRNA was calculated as a sum of the copy number of the reference sequence and the sequences mapping at the same 5'-end position as the reference sequence. The expression level of each miRNA was calculated as the Transcripts Per Million reads (TPM): the number of reads mapped to the transcript normalized by the number of total mapped reads.

### miRNA binding site extraction from *in silico* prediction

Human miRNA target prediction was based on miRNA target site identified using Targetscan [33] and additional target site conservation criterion. The mature miRNA sequences were downloaded from miRBase (v17). The 3'UTR sequences of human, mouse, rat, dog and chicken were downloaded from Ensemble Genes (v64) using biomart. The 3'UTR sequence alignments of five species was conducted using MUSCLE [34] with default parameters, aside from requiring the "stable" parameter to keep the input sequence order. Only the alignments with less than 50% of the gap sequence in human, mouse, rat, dog and chicken were used for downstream analysis. The scripts from the TargetScan website (v5.2) were used to predict miRNA target sites across five species. Only target sites that were conserved in at least 3 out of 4 species (mouse, rat, dog and chicken) were classified as reliable conserved targets.

These results were compared to target predictions obtained by tools based on different prediction strategies in downstream analysis, with mirDB [1] (version 3.0) based on support vector machines (SVMs) trained by microarray experiment data, and CoMeTa [2] based on co-expression meta-analysis ranking pooled targets predicted by multiple algorithms.

### Enriched targeting miRNA identification

To identify miRNA with enrichment of predicted targets in discordant gene groups in each of the four patterns, we compared miRNA binding site number with concordant genes in the same pattern. To avoid the influence of miRNA gene families, we restricted this comparison to a single test per unique miRNA seed. If a miRNA's predicted targets were enriched in a pattern at hypergeometric test $p < 0.05$ after Bonferroni correction, we considered that miRNA "specific" to that discordant gene group. All the other miRNAs were considered "non-specific" between the concordant and discordant gene groups in this pattern. These results were compared to chance distributions obtained using 1,000 permutations by randomized labeling of concordant/discordant genes within each pattern.

### Functional miRNA regulation estimation

To estimate the negative effect of miRNAs on the translation of discordant genes in each pattern, we calculated the predicted miRNA-target distribution of the Spearman's rank correlation coefficients measured based on expression profiles of age-dependent miRNA and protein expression of their predicted target transcripts in aging interval.

We estimated the background chance distribution by generating the same number of predicted miRNA-target pairs based on randomly chosen age-dependent miRNA and target genes within the discordant group of each pattern for 1,000 times.

We then estimated the difference between predicted miRNA-target distribution and background chance distribution by subtraction of the median of background chance distribution. A discordant

group of one pattern was considered negatively regulated by miRNAs on the translation level, if and only if significant excess of negative correlation ($p < 0.05$, FDR < 5%, Spearman's rank correlation) was observed. The significance is indicated by 95% confident intervals of chance difference obtained in background chance distribution.

## Supplemental References


1. Wang, X. (2008). miRDB: a microRNA target prediction and functional annotation database with a wiki interface. Rna *14*, 1012-1017.

2. Gennarino, V.A., D'Angelo, G., Dharmalingam, G., Fernandez, S., Russolillo, G., Sanges, R., Mutarelli, M., Belcastro, V., Ballabio, A., Verde, P., et al. (2012). Identification of microRNA-regulated gene networks by expression analysis of target genes. Genome research *22*, 1163-1172.

3. Kishore, S., Jaskiewicz, L., Burger, L., Hausser, J., Khorshid, M., and Zavolan, M. (2011). A quantitative analysis of CLIP methods for identifying binding sites of RNA-binding proteins. Nature methods *8*, 559-564.

4. Hoell, J.I., Larsson, E., Runge, S., Nusbaum, J.D., Duggimpudi, S., Farazi, T.A., Hafner, M., Borkhardt, A., Sander, C., and Tuschl, T. (2011). RNA targets of wild-type and mutant FET family proteins. Nature structural & molecular biology *18*, 1428-1431.

5. Ascano, M., Jr., Mukherjee, N., Bandaru, P., Miller, J.B., Nusbaum, J.D., Corcoran, D.L., Langlois, C., Munschauer, M., Dewell, S., Hafner, M., et al. (2012). FMRP targets distinct mRNA sequence elements to regulate protein expression. Nature *492*, 382-386.

6. Konig, J., Zarnack, K., Rot, G., Curk, T., Kayikci, M., Zupan, B., Turner, D.J., Luscombe, N.M., and Ule, J. (2010). iCLIP reveals the function of hnRNP particles in splicing at individual nucleotide resolution. Nature structural & molecular biology *17*, 909-915.

7. Hafner, M., Landthaler, M., Burger, L., Khorshid, M., Hausser, J., Berninger, P., Rothballer, A., Ascano, M., Jr., Jungkamp, A.C., Munschauer, M., et al. (2010). Transcriptome-wide identification of RNA-binding protein and microRNA target sites by PAR-CLIP. Cell *141*, 129-141.

8. Sievers, C., Schlumpf, T., Sawarkar, R., Comoglio, F., and Paro, R. (2012). Mixture models and wavelet transforms reveal high confidence RNA-protein interaction sites in MOV10 PAR-CLIP data. Nucleic acids research *40*, e160.

9. Sanford, J.R., Wang, X., Mort, M., Vanduyn, N., Cooper, D.N., Mooney, S.D., Edenberg, H.J., and Liu, Y. (2009). Splicing factor SFRS1 recognizes a functionally diverse landscape of RNA transcripts. Genome research *19*, 381-394.

10. Tollervey, J.R., Curk, T., Rogelj, B., Briese, M., Cereda, M., Kayikci, M., Konig, J., Hortobagyi, T., Nishimura, A.L., Zupunski, V., et al. (2011). Characterizing the RNA targets and position-dependent splicing regulation by TDP-43. Nature neuroscience *14*, 452-458.

11. Wang, Z., Kayikci, M., Briese, M., Zarnack, K., Luscombe, N.M., Rot, G., Zupan, B., Curk, T., and Ule, J. (2010). iCLIP predicts the dual splicing effects of TIA-RNA interactions. PLoS biology *8*, e1000530.

12. Schwartz, S., Kent, W.J., Smit, A., Zhang, Z., Baertsch, R., Hardison, R.C., Haussler, D., and Miller, W. (2003). Human-mouse alignments with BLASTZ. Genome research *13*, 103-107.

13. Mazin, P., Xiong, J., Liu, X., Yan, Z., Zhang, X., Li, M., He, L., Somel, M., Yuan, Y., Phoebe Chen, Y.P., et al. (2013). Widespread splicing changes in human brain development and aging. Molecular systems biology *9*, 633.

14. Dobin, A., Davis, C.A., Schlesinger, F., Drenkow, J., Zaleski, C., Jha, S., Batut, P., Chaisson, M., and Gingeras, T.R. (2013). STAR: ultrafast universal RNA-seq aligner. Bioinformatics *29*, 15-21.

15. Li, H., Handsaker, B., Wysoker, A., Fennell, T., Ruan, J., Homer, N., Marth, G., Abecasis, G., Durbin, R., and Genome Project Data Processing, S. (2009). The Sequence Alignment/Map format and SAMtools. Bioinformatics *25*, 2078-2079.

16. Harrow, J., Frankish, A., Gonzalez, J.M., Tapanari, E., Diekhans, M., Kokocinski, F., Aken, B.L., Barrell, D., Zadissa, A., Searle, S., et al. (2012). GENCODE: the reference human genome annotation for The ENCODE Project. Genome research *22*, 1760-1774.

17. UniProt, C. (2014). Activities at the Universal Protein Resource (UniProt). Nucleic acids research *42*, D191-198.

18. Granholm, V., Kim, S., Navarro, J.C., Sjolund, E., Smith, R.D., and Kall, L. (2014). Fast and accurate database searches with MS-GF+Percolator. Journal of proteome research *13*, 890-897.

19. Zhu, W., Smith, J.W., and Huang, C.M. (2010). Mass spectrometry-based label-free quantitative proteomics. Journal of biomedicine & biotechnology *2010*, 840518.

20. Clancy, B., Darlington, R.B., and Finlay, B.L. (2001). Translating developmental time across mammalian species. Neuroscience *105*, 7-17.

21. Rodwell, G.E., Sonu, R., Zahn, J.M., Lund, J., Wilhelmy, J., Wang, L., Xiao, W., Mindrinos, M., Crane, E., Segal, E., et al. (2004). A transcriptional profile of aging in the human kidney. PLoS biology *2*, e427.

22. Erraji-Benchekroun, L., Underwood, M.D., Arango, V., Galfalvy, H., Pavlidis, P., Smyrniotopoulos, P., Mann, J.J., and Sibille, E. (2005). Molecular aging in human prefrontal cortex is selective and continuous throughout adult life. Biological psychiatry *57*, 549-558.

23. Somel, M., Franz, H., Yan, Z., Lorenc, A., Guo, S., Giger, T., Kelso, J., Nickel, B., Dannemann, M., Bahn, S., et al. (2009). Transcriptional neoteny in the human brain. Proceedings of the National Academy of Sciences of the United States of America *106*, 5743-5748.

24. de Magalhaes, J.P., and Costa, J. (2009). A database of vertebrate longevity records and their relation to other life-history traits. Journal of evolutionary biology *22*, 1770-1774.

25. Walker, R., Gurven, M., Hill, K., Migliano, A., Chagnon, N., De Souza, R., Djurovic, G., Hames, R., Hurtado, A.M., Kaplan, H., et al. (2006). Growth rates and life histories in twenty-two small-scale societies. American journal of human biology : the official journal of the Human Biology Council *18*, 295-311.

26. Lu, T., Pan, Y., Kao, S.Y., Li, C., Kohane, I., Chan, J., and Yankner, B.A. (2004). Gene regulation and DNA damage in the aging human brain. Nature *429*, 883-891.

27. Kanehisa, M., Goto, S., Sato, Y., Kawashima, M., Furumichi, M., and Tanabe, M. (2014). Data, information, knowledge and principle: back to metabolism in KEGG. Nucleic acids research *42*, D199-205.

28. Reference Genome Group of the Gene Ontology, C. (2009). The Gene Ontology's Reference Genome Project: a unified framework for functional annotation across species. PLoS computational biology *5*, e1000431.

29. Tabas-Madrid, D., Nogales-Cadenas, R., and Pascual-Montano, A. (2012). GeneCodis3: a non-redundant and modular enrichment analysis tool for functional genomics. Nucleic acids research *40*, W478-483.

30. Somel, M., Guo, S., Fu, N., Yan, Z., Hu, H.Y., Xu, Y., Yuan, Y., Ning, Z., Hu, Y., Menzel, C., et al. (2010). MicroRNA, mRNA, and protein expression link development and aging in human and macaque brain. Genome research *20*, 1207-1218.

31. Hu, H.Y., Yan, Z., Xu, Y., Hu, H., Menzel, C., Zhou, Y.H., Chen, W., and Khaitovich, P. (2009). Sequence features associated with microRNA strand selection in humans and flies. BMC genomics *10*, 413.

32. Langmead, B., Trapnell, C., Pop, M., and Salzberg, S.L. (2009). Ultrafast and memory-efficient alignment of short DNA sequences to the human genome. Genome biology *10*, R25.

33. Lewis, B.P., Burge, C.B., and Bartel, D.P. (2005). Conserved seed pairing, often flanked by adenosines, indicates that thousands of human genes are microRNA targets. Cell *120*, 15-20.

34. Edgar, R.C. (2004). MUSCLE: multiple sequence alignment with high accuracy and high throughput. Nucleic acids research *32*, 1792-1797.
